# Global transcriptome analysis profiles metabolic pathways in traditional herb *Astragalus membranaceus *Bge. var. *mongolicus *(Bge.) Hsiao

**DOI:** 10.1186/1471-2164-16-S7-S15

**Published:** 2015-06-11

**Authors:** Jing Chen, Xue-Ting Wu, Yi-Qin Xu, Yang Zhong, Yi-Xue Li, Jia-Kuan Chen, Xuan Li, Peng Nan

**Affiliations:** 1 Ministry of Education Key Laboratory for Biodiversity Science and Ecological Engineering, School of Life Sciences, Fudan University, Shanghai 200438, China; 2 Key Laboratory of Synthetic Biology, Institute of Plant Physiology and Ecology, Shanghai Institutes for Biological Sciences, Chinese Academy of Sciences, Shanghai 200032, China; 3 Institute of Biodiversity Science and Geobiology, Tibet University, Lhasa 850000, China; 4 Shanghai Center for Bioinformation Technology, Shanghai Academy of Science and Technology, Shanghai 201203, China

**Keywords:** *Astragalus membranaceus *Bge. var. *mongolicus *(Bge.) Hsiao, Transcriptome, Herb, Metabolites, Isoflavonoids, Triterpene saponins

## Abstract

**Background:**

*Astragalus membranaceus *Bge. var. *mongolicus *(Bge.) Hsiao (*A. mongolicus*, family Leguminosae) is one of the most important traditional Chinese herbs. Among many secondary metabolites it produces, the effective bioactive constituents include isoflavonoids and triterpene saponins. The genomic resources regarding the biosynthesis of these metabolites in *A. mongolicus *are limited. Although roots are the primary material harvested for medical use, the biosynthesis of the bioactive compounds and its regulation in *A. mongolicus *are not well understood. Therefore, a global transcriptome analysis on *A. mongolicus *tissues was performed to identify the genes essential for the metabolism and to profile their expression patterns in greater details.

**Results:**

RNA-sequencing was performed for three different *A. mongolicus *tissues: leaf, stem, and root, using the Illumina Hiseq2000 platform. A total of 159.5 million raw sequence reads were generated, and assembled into 186,324 unigenes with an N50 of 1,524bp. Among them, 129,966 unigenes (~69.7%) were annotated using four public databases (Swiss-Prot, TrEMBL, CDD, Pfam), and 90,202, 63,946, and 78,326 unigenes were found to express in leaves, roots, and stems, respectively. A total of 8,025 transcription factors (TFs) were identified, in which the four largest families, bHLH, MYB, C3H, and WRKY, were implicated in regulation of tissue development, metabolisms, stress response, etc. Unigenes associated with secondary metabolism, especially those with isolavonoids and triterpene saponins biosynthesis were characterized and profiled. Most genes involved in the isoflavonoids biosynthesis had the lowest expression in the leaves, and the highest in the stems. For triterpene saponin biosynthesis, we found the genes in MVA and non-MVA pathways were differentially expressed among three examined tissues, indicating the parallel but compartmentally separated biosynthesis pathways of IPP and DMAPP in *A. mongolicus*. The first committed enzyme in triterpene saponin biosynthesis from *A. mongolicus*, cycloartenol synthase (AmCAS), which belongs to the oxidosqualene cyclase family, was cloned by us to study the astragalosides biosynthesis. Further co-expression analysis indicated the candidate CYP450s and glycosyltransferases (GTs) in the cascade of triterpene saponins biosynthesis. The presence of the large CYP450 families in *A. mongolicus *was further compared with those from *Medicago truncatula *and *Arabidopsis thaliana*, and the diversity and phylegenetic relationships of the CYP450 families were established.

**Conclusion:**

A transcriptome study was performed for *A. mongolicus *tissues to construct and profile their metabolic pathways, especially for the important bioactive molecules. The results revealed a comprehensive profile for metabolic activities among tissues, pointing to the equal importance of leaf, stem, and root in *A. mongolicus *for the production of bioactive compounds. This work provides valuable resources for bioengineering and in vitro synthesis of the natural compounds for medical research and for potential drug development.

## Background

*Astragalus membranaceus *Bge. var. *mongolicus *(Bge.) Hsiao (*A. mongolicus*) is a perennial herbaceous plant that belongs to the legume family. The dried root of this plant, known as "Huangqi", is one of the most commonly used traditional Chinese herbs. *A. mongolicus *has a growth cycle of 2 to 3 years, and its root is often harvested in the spring or autumn, before being dried under the sun. Being an important ingredient for Chinese medicine for thousands of years, *A. mongolicus *is used to reinforce vital energy and body immunity. It's also used as an antiperspirant, a diuretic, and often recommended as an adjuvant drug during cancer and diabetes therapy [1]. Polysaccharide, triterpenes, flavonoids, zinc, molybdenum, silicon and other trace elements are regarded as its effective components, of which calycosin, calycosin-7-O-β-D-glucoside (CG) and astragaloside IV(ASI) are major compounds related to the bioactivity of the herb[2-4].

Isoflavones are a subclass of phenylpropanoid secondary metabolites produced mainly in legumes, with a wide range of biological activities. Calycosin and CG are the major isoflavones of effective constituents in *A. mongolicus*[3]. Both calycosin and CG are supposed to be natural anti-inflammatory products[5]. In addition, calycosin could be used to improve body immunity, anti-radiation, anti-cancer, anti-microbial and protect endothelial cells from hypoxia-induced barrier impairment. It is also considered to act as antioxidants and adjuvant drugs for reducing blood lipid and glucose[6]. CG could be used as an anti-osteoarthritis medicine, a hyaluronidase inhibitory component and has the effect to protect from proteoglycan degradation [7,8]. Calycosin and CG are synthesized from L-phenylalanine via the isoflavonoids branch of the phenylpropanoid metabolism[9].The biosynthetic pathway of calycosin and CG in legumes has been documented previously[10,11].

Triterpenoid saponins are a class of important secondary metabolites in plants, which has various usages as adjuvants, sweeteners, foaming agents, precursors for hormone synthesis, and so on[12]. They are considered to be the major bioactive compounds responsible for the pharmacological features in many valued Chinese traditional herbs, such as *Panax notoginseng, Panax ginseng *and *Panax quinquefolius *L [13-15]. ASI is one of such natural triterpenoid saponins isolated from *A. mongolicus*, and has been used for the quality evaluation of *A. mongolicus*[1]. As a major effective constituent, ASI shows rich pharmacologic activities including anti-fatigue, anti-cancer, anti-coxsackie B virus, etc[16].

Triterpenoid saponins are synthesized from the universal terpenoid precursors, isopentenyl diphosphate (IPP) and dimethylallyl diphosphate (DMAPP). In plants, IPP and DMAPP either derive from condensation of acetyl-CoA in the cytosolic mevalonate pathway or from pyruvate and phosphoglyceraldehyde in the plastidial non-MVA(MEP/DXP) pathway[17]. Downstream formation of triterpenoids is a complex multi-step process, including chain elongation, isomerization, cyclization, chain coupling, epoxide protonation, oxidation and glycosylation. In recent years, the classification, pharmacological activity and biosynthesis of ginsenosides, a kind of triterpenoid from *Panax *species, have been studied extensively. To date, at least 150 natural ginsenosides have been isolated from *Panax *species, and more than 20 genes related to the synthesis of ginsenosides have been cloned[15,18]. In comparision with *Panax *species, less than 20 triterpenoid saponins were isolated in *A. mongolicus*. Most researches focus on the bioactivity of *A. mongolicus *as traditional herb, and there are few reports about biosynthesis pathway of ASI[19].

In spite of its pharmacological importance, the genomic data of *A. mongolicus *is very limited. So far, only 271 nucleotide sequences and 11 ESTs are available in the National Center for Biotechnology Information (NCBI) databases. The limited data hamper the research of active ingredients biosynthetic and metabolic mechanisms in *A. mongolicus*. In recent years, RNA-seq is quickly developed as a massively parallel sequencing method to generate transcriptome profiles at large scale and relatively low cost[20,21]. Since RNA-seq is not restricted to detecting transcripts of known genes, it is particularly useful for non-model organisms with no available genomic data[22]. Over the past several years, RNA-seq were applied to generate large scale transcriptomic data in non-model plant species such as *Camellia sinensis*[23,24], *Panax notoginseng*[13], and *Panax quinquefolius*[15].

Recently, a study of *Astragalus membranaceus *(Fisch.) Bge used 454 GS FLX technology to generate an expressed sequence tag (EST) dataset from leaves of *Astragalus membranaceus*[25]. To comprehensively compare transcriptional profiles among tissues, our study was designed to generate the transcriptome of *A. mongolicus *tissues (leaf, stem and root), profile the important metabolic pathways in *A. mongolicus*, and identify and characterize novel genes for the synthesis of bioactive compounds. Our work produced a total of 159.5 million sequence reads (15.9 Gbp), resulting in 186,324 assembled unigenes. The results of current work reveals comprehensive profiles of metabolic activities among *A. mongolicus *tissues, and will serve as a valuable public resource facilitating the research on traditional herbs and development of natural bioactive products for therapeutic needs.

## Results and discussion

### Sample collection, and sequencing

**Figure 1:**
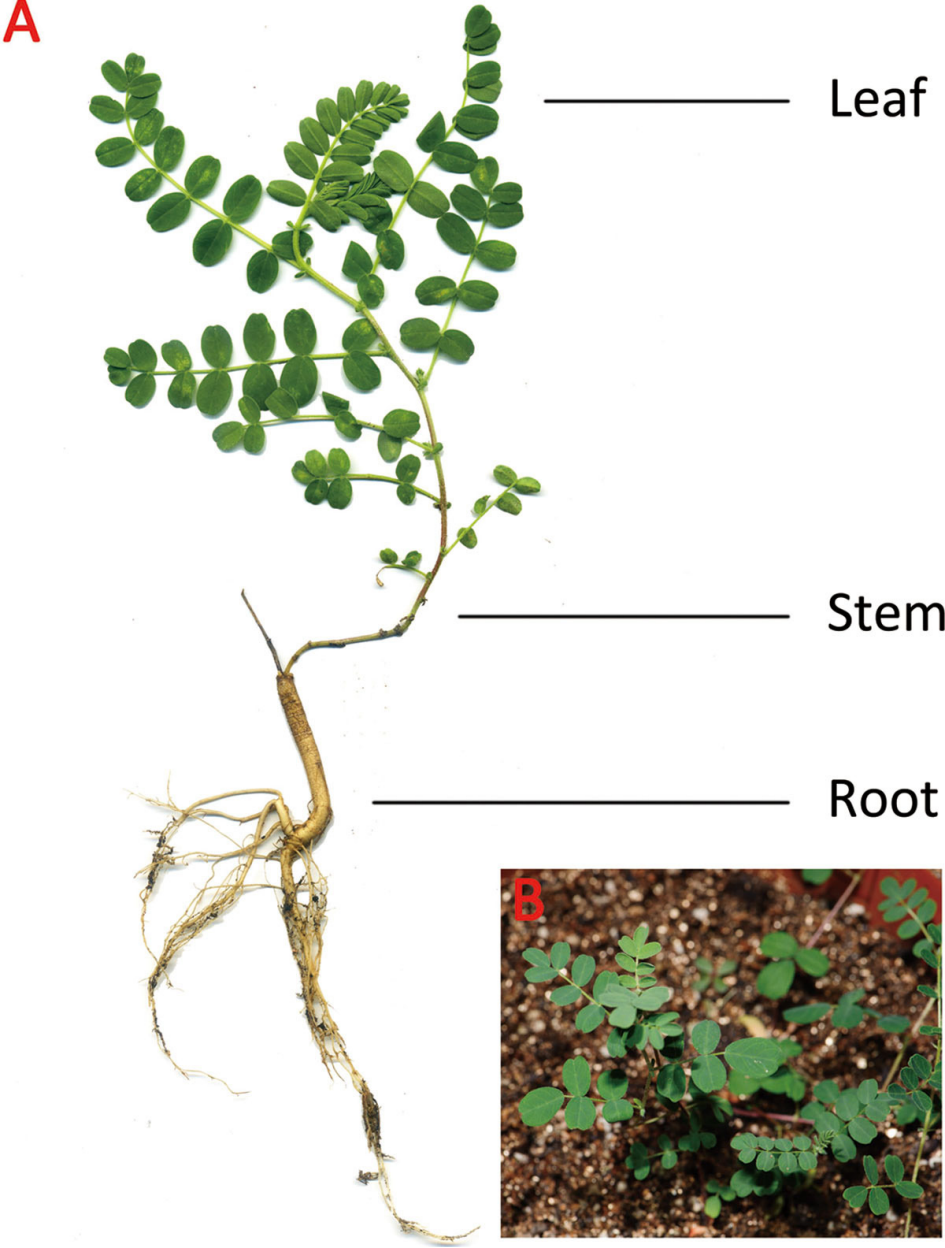
***A. mongolicus *used in this study**. A) whole-plant. The name of each tissue was labeled with black words. B) *A. mongolicus *in normal growth conditions.

Roots are the main medicinal materials from *A. mongolicus *with abundant bioactive ingredients. However, previous study showed that accumulation of calycosin and CG are regulated by different mechanisms in various tissues[9]. To generate *A. mongolicus *transcriptome and compare transcriptional profiles among tissues, total RNA was extracted from three different sample tissues: leaf, stem, and root (Figure 1). RNA-seq libraries were prepared from the collected tissues, and Illumina sequencing was performed on each RNA-seq library by Illummina HiSeq 2500 system, with a pair-end read length of 100 base pairs (bp). A total of 159.5 million raw reads (approximate 15.9Gbp) were obtained for all three *A. mongolicus *tissues, whereas those from leaves, roots, and stems were 4.7Gbp, 5.2Gbp and 5.9Gbp, respectively (Table 1). Since adaptor sequences, ambiguous reads and low-quality reads may lead to erroneous assemblies, we removed the low-quality or ambiguous nucleotides from both ends of the reads, resulting in 154.4 million high-quality reads (15.2 Gbp) with an average length of 98.84 bp.

**Table 1:** Overview of the sequencing and assembly of transcriptome of *A. mongolicus*.

	No. of reads	No. of bases(bp)
Leaf	47,587,040	4,758,704,000
root	52,497,000	5,249,700,000
stem	59,385,462	5,938,546,200
Total raw data	159,469,502	15,946,950,200
Total high-quality data	154,439,892	15,264,422,138
Average high-quality read length (bp)	98.84	
Average length of unigenes (bp)	895.05	
Range of unigenes length (bp)	201-13,149	
Unigenes > = 200 bp	186,324	166,768,907
N50 (bp)	1,524	

### Transcriptome assembly and gene expression comparison among tissues

**Figure 2:**
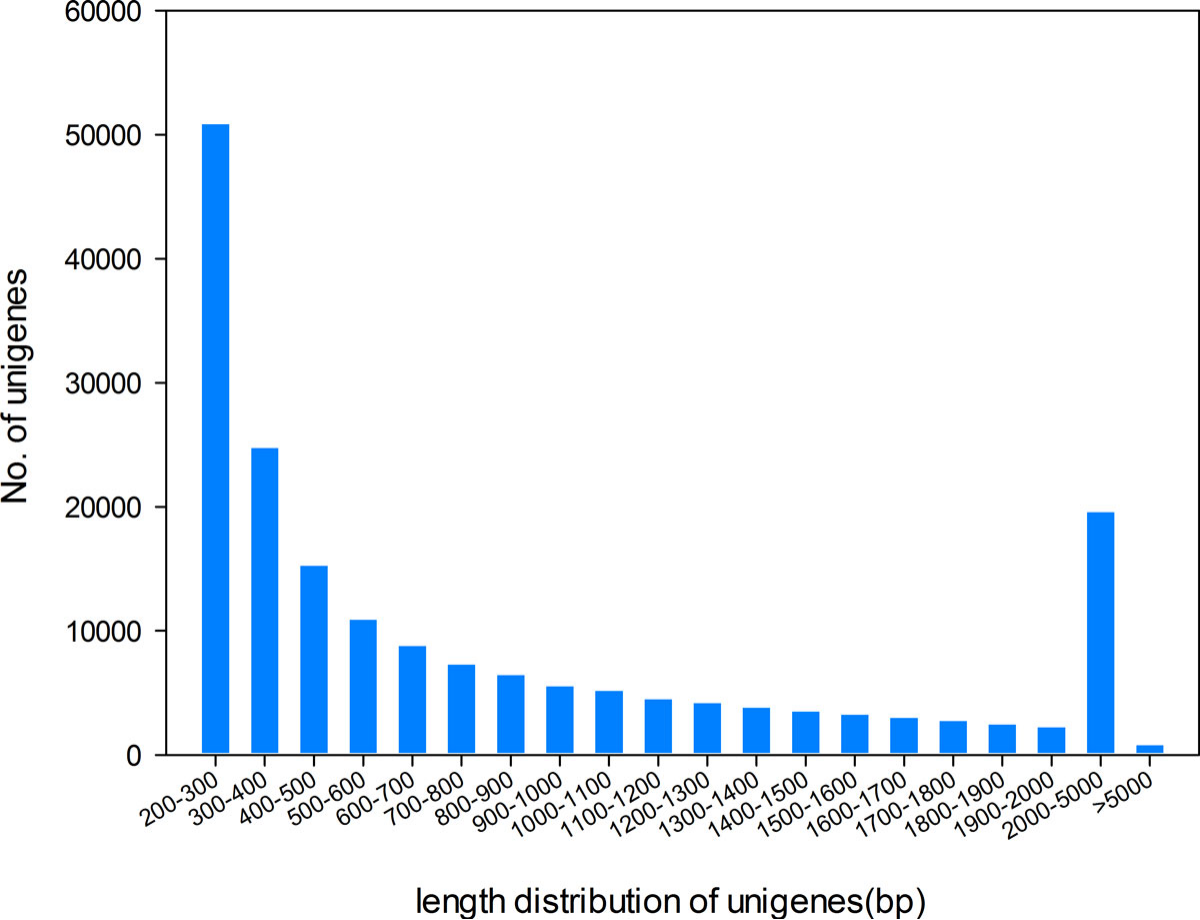
**Length distribution of assembled unigenes**. Out of the 186,324 unigenes, 95,274 unigenes (51.1%) were longer than 500 bp and 55,988 (30%) were longer than 1000 bp

High-quality reads from the three tissues were combined together and de novo assembly was performed with Trinity (release 20130225) [26], which was specially developed for de novo assembly from short-read RNA-Seq data[24]. Hence, 186,324 unigenes was obtained, ranging from 201 bp to 13,149 bp in size, with a total size of 166.7 Mbp. The average length of the assembled transcripts was 895 bp, whereas their N50 is 1,524 bp. The length distribution of unigenes was shown in Figure 2, in which 95,274 unigenes (51.1%) were longer than 500 bp and 55,988 (30%) were longer than 1000 bp.

**Figure 3:**
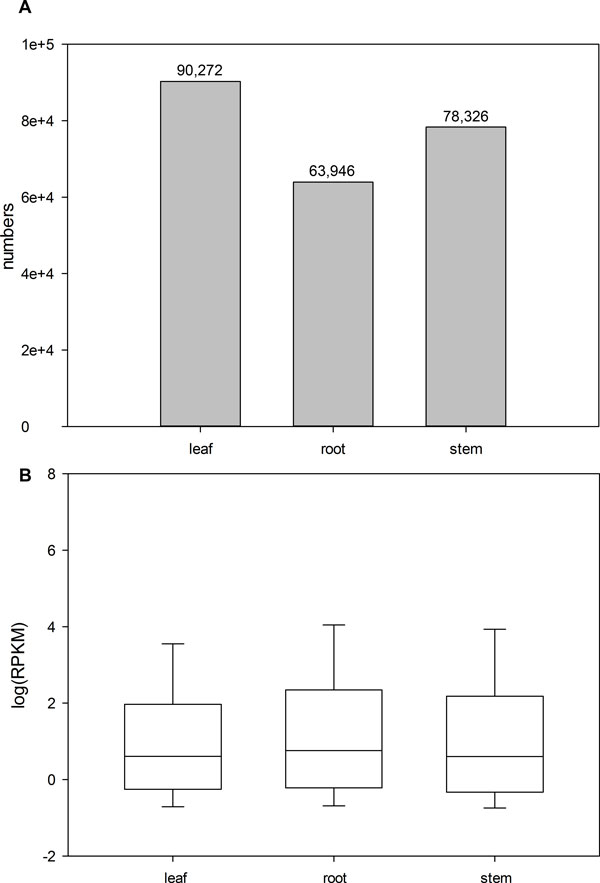
**Unigenes expressed number and level in different tissues**. A) The number of expressed genes (RPKM ≥ 0.5) in each tissue was labeled over the bars. B) Expressional levels of unigenes of three tissues from *A. mongolicus*. We took the logarithm of each RPKM value of all unigenes.

In order to get the gene expression profiles of each individual tissue, the preprocessed reads (clean reads) from the three tissues were mapped back to the assembled unigenes using bowtie2[27]. To assess the gene expression abundance, the expressional levels of unigenes were measured by RPKM values, with RPKM ≥ 0.5 used as a cut-off. The number of expressed unigenes and distribution of expression levels in each tissue are shown in Figure 3. While the leaf tissue has the highest numbers of gene expressed, all the three tissues shared a similar distribution in gene expression level.

**Figure 4:**
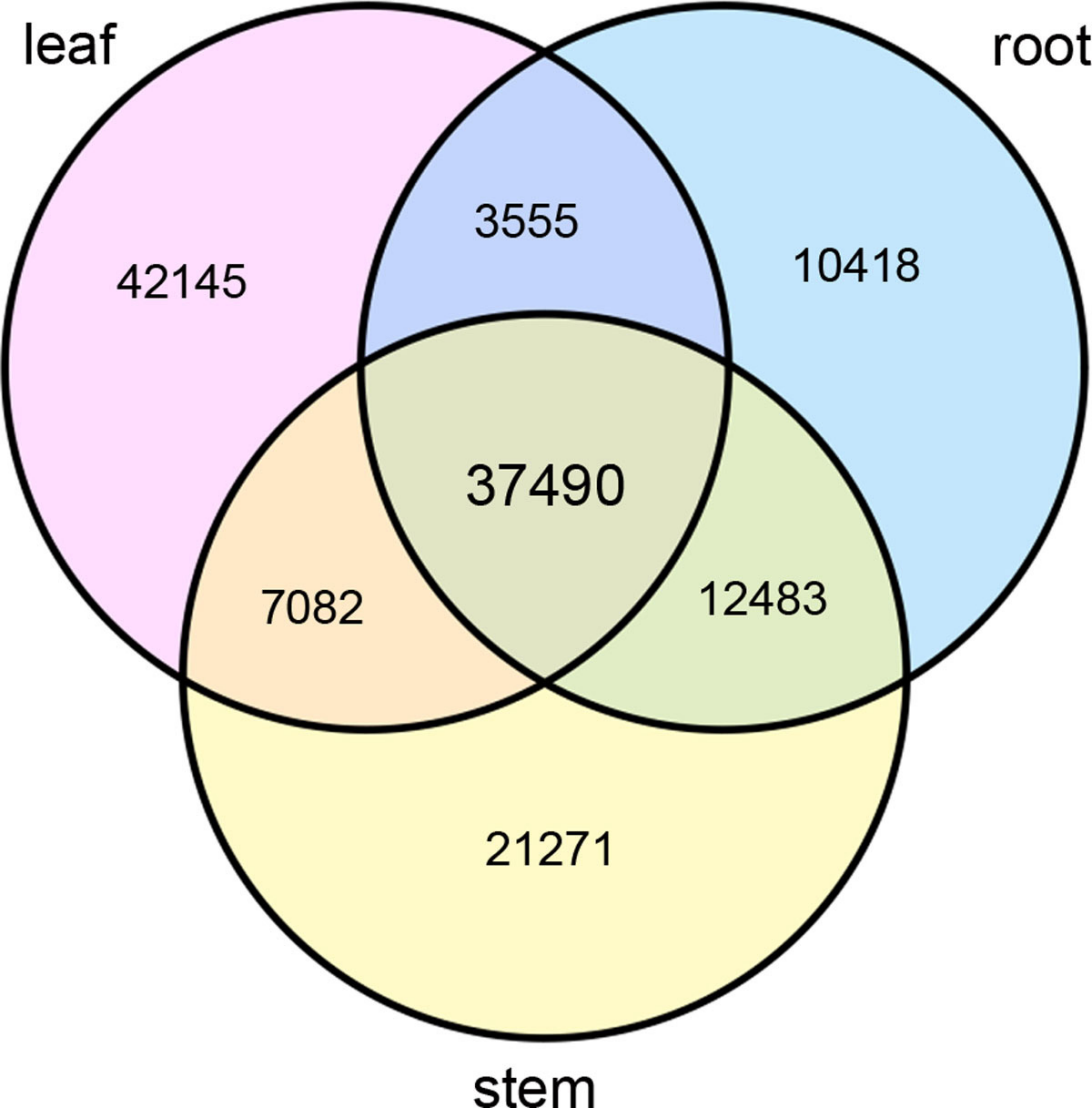
**Venn diagram of expressed unigenes in three tissues**. A total of 134,445 unigenes (72.15%) were expressed, of which 37,490 genes were common expressed in all the three tissues. Unigenes with RPKM≥0.5 were considered as expressed in each tissue.

The differentially expressed unigenes in the three tissues were investigated. There were in total 134,445 unigenes (72.15%) expressed above the threshold level (RPKM ≥ 0.5) in three tissues, of which 37,490 genes were common expressed in all the three tissues (Figure 4). In addition, some expressed genes were shared by two of the three tissues. The root and stem shared the most expressed unigenes (12,483), followed by the leaf and stem (7082). The leaf expressed the largest number of tissue-specific unigenes (42,145). The root, as the most commonly used part for medicinal material, expressed 104,18 tissue-specific unigenes, of which 5,209 are considered highly expressed (> = 0.83 RPKM). These and other genes up-regulated in the root probably contribute significantly to the utility of root in therapeutic practice. Many of these genes, i.e. Phenylalanine ammonia lyase (*PAL*), cinnamate-4-hydroxylase (*C4H*), hydroxymethylglutaryl-CoA synthase (*HMGCS*), mevalonate kinase (*MVK*) and diphosphomevalonate decarboxylase (*MVD*) are found to participate in synthesis pathways for secondary metabolites, including isoflavonoids and triterpenoids.

### Functional annotation of transcriptome for *A. mongolicus*

For the purpose of predicting and analyzing the function of assembled unigenes, all assembled unigenes were annotated based on sequence similarity using four public databases, including Swiss-Prot protein database (Swiss-Prot), the Translated EMBL Nucleotide Sequence database (TrEMBL), the Conserved Domain Database (CDD) and Pfam database. In total, 69.75% of all unigenes had significant hits in the four databases (71,708 unigenes annotated with the Swiss-Prot, 104,568 with the TrEMBL, 114,904 with the CDD, and 76,680 with pfamA) (Table 2, Additional file 1). The remaining 30.25% of unigenes had no significant matches in any of the public databases, which indicates these novel genes are specific to *A. mongolicus*. These species-specific genes have unknown function, which are of great interest to be further characterized in the future.

**Table 2:** Summary of unigenes annotation.

Database	Total Unigenes	Annotated Unigenes	Percent	Unannotated Unigenes	Percent
Swiss-Prot	186,324	71,708	38.49%	114,616	61.51%
CDD	186,324	114,904	61.67%	71,420	38.33%
TrEMBL	186,324	104,568	56.12%	81,756	43.88%
pfamA	186,324	76,680	41.15%	110,366	59.23%
Total	186,324	129,966	69.75%	56,358	30.25%

**Figure 5:**
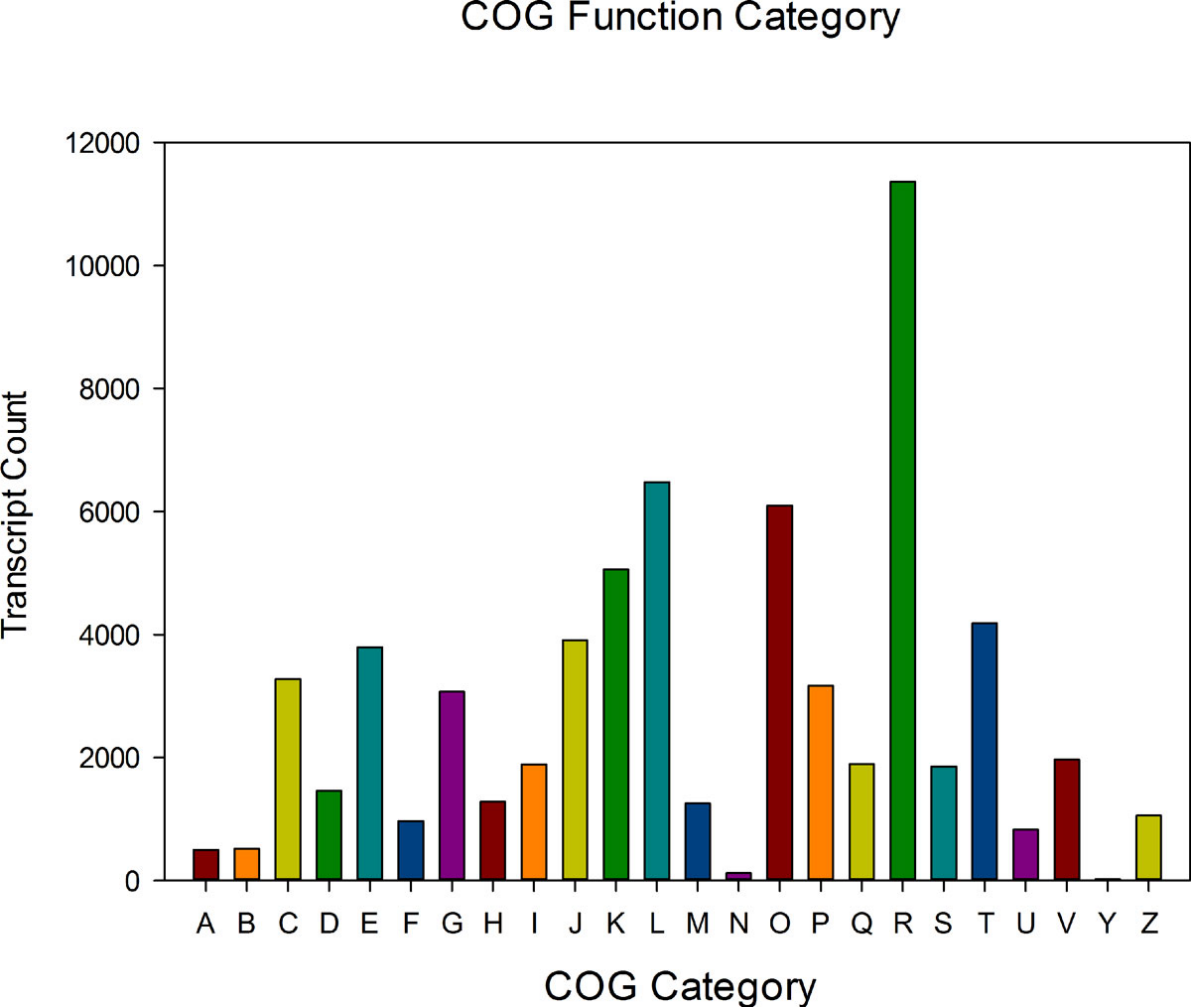
**COG function classifications of unigenes**. A total of 65,989 unigenes were classified into 24 COG categories. A: RNA processing and modification; B: Chromatin structure and dynamics; C: Energy production and conversion; D: Cell cycle control, cell division, chromosome partitioning; E: Amino acid transport and metabolism; F: Nucleotide transport and metabolism; G: Carbohydrate transport and metabolism; H: Coenzyme transport and metabolism; I: Lipid transport and metabolism; J: Translation, ribosomal structure and biogenesis; K: Transcription; L: Replication, recombination and repair; M: Cell wall/membrane/envelope biogenesis; N: Cell motility; O: Posttranslational modification, protein turnover, chaperones; P: Inorganic ion transport and metabolism; Q: Secondary metabolites biosynthesis, transport and catabolism; R: General function prediction only; S: Function unknown; T: Signal transduction mechanisms; U: Intracellular trafficking, secretion, and vesicular transport; V: Defense mechanisms; Y: Nuclear structure; Z: Cytoskeleton.

Cluster of orthologous groups (COG) consists of protein sequences from both prokaryotic and eukaryotic genomes (bacteria, algae and eukaryotes), which were classified based on their phylogenetic relationship[28]. Phylogenetic classifications of the conserved domains of unigenes from *A. mongolicus *were analyzed by BLAST searching against COG database to predict and classify possible functions. In our study, 65,989 unigenes were classified into 24 COG categories (Figure 5). The top five categories include 1) General function prediction only (17.22%, functions associated with basic physiological and metabolic functions); 2) Replication, recombination and repair (9.82%); 3) Posttranslational modification, protein turnover, chaperones (9.24%); 4) Transcription (7.66%); and 5) Signal transduction mechanisms (6.34%)(Additional file 2). Unigenes involved in secondary metabolism (secondary metabolite biosynthesis, transport and catabolism) occupies about 2.87% (1,896 transcripts) of all COG-annotated transcripts.

A database resource for studying functions of a biological system, Kyoto Encyclopedia of Genes and Genomes (KEGG) collected pathway information on the molecular interaction and reaction networks, which helps conduct pathway-based analysis to study genes of unknown biological systems. To systematically investigate the metabolic pathways and related biological features, the unigenes from *A. mongolicus *were annotated against the KEGG database using the KAAS online tool (http://www.genome.jp/tools/kaas). As a result, 13,057 transcripts were annotated with Enzyme Commission (EC) numbers and assigned to 339 reference canonical KEGG pathways (Additional file 3). Among the unigenes assigned to the KEGG pathways, 130 of them were assigned to 'Ribosome', followed by 'Oxidative phosphorylation'(116), 'Biosynthesis of amino acids' (114), 'Purine metabolism' (114), Huntington's disease (108), and others. Overall, these annotation analyses are helpful for predicting the molecular functions of the unigenes, and reconstructing the metabolic pathways in *A. mongolicus*.

### Discovery of putative transcription factors in *A. mongolicus*

**Figure 6:**
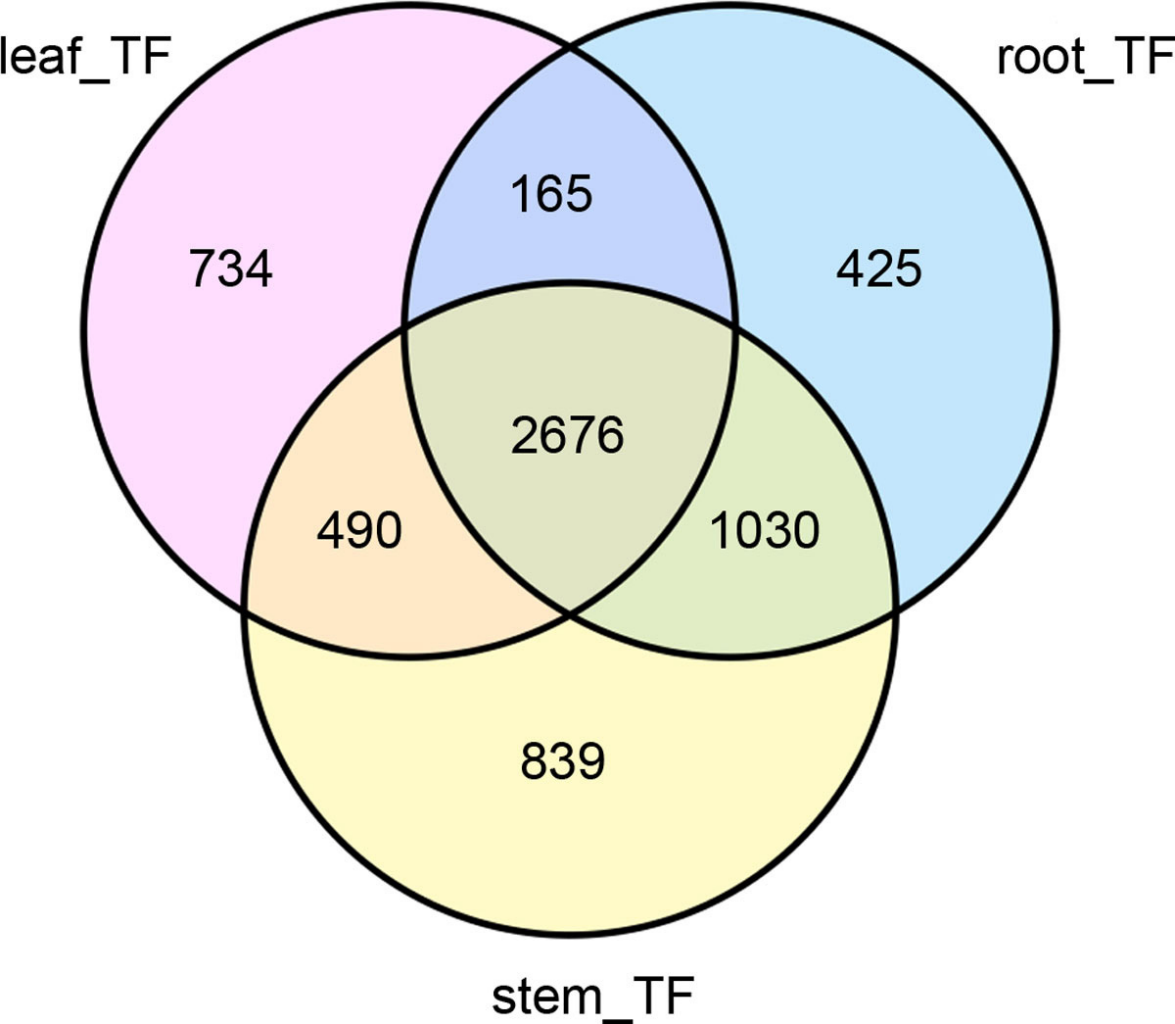
**Venn diagram showing expressed TFs in three tissues**. A total of 6,359 TFs (79.23%) were significantly expressed, of which 2,676 TFs were commonly expressed in all three tissues. Unigenes with RPKM≥0.5 were considered as expressed in each tissue.

**Figure 7:**
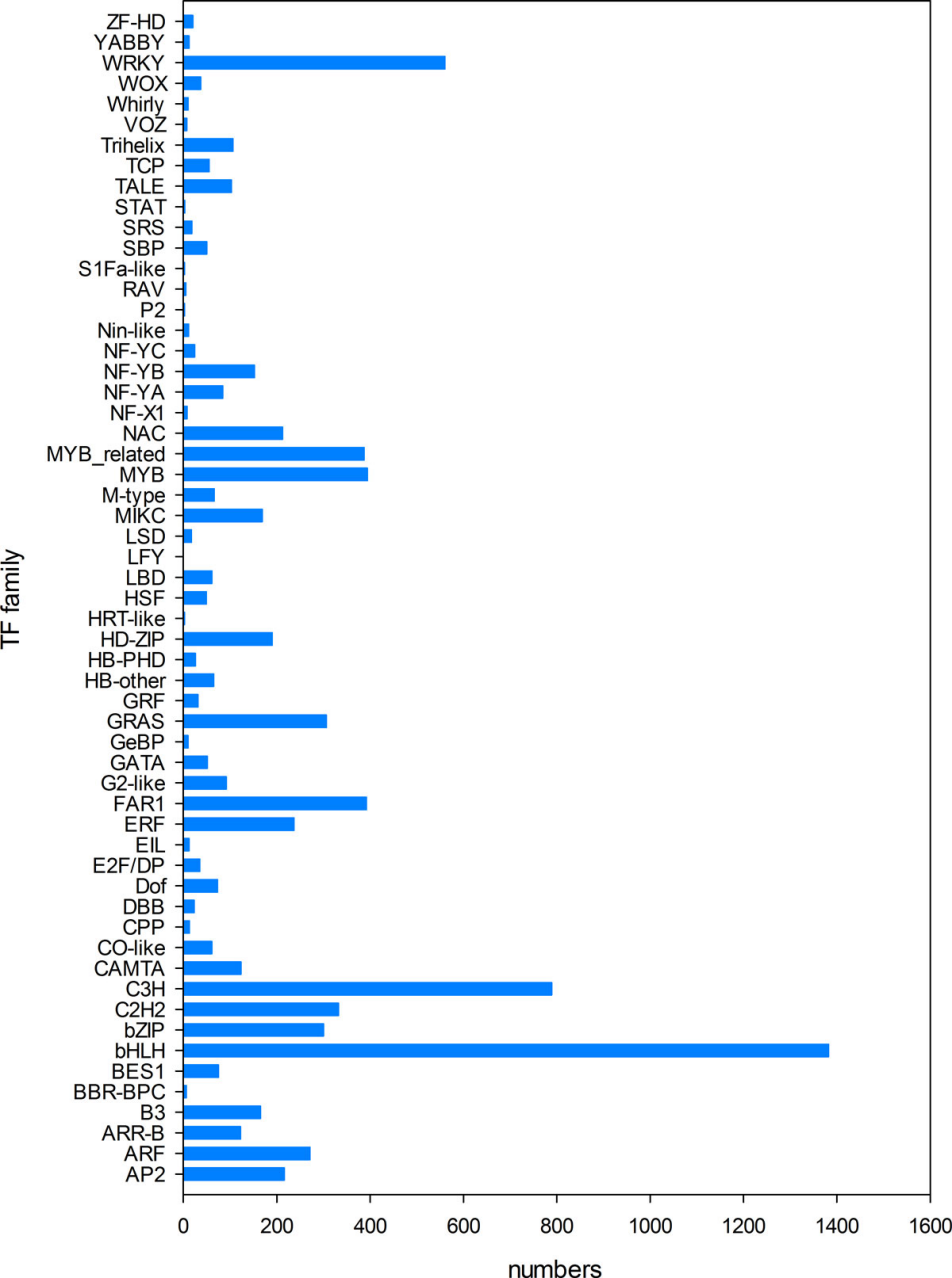
**Classification of TF families**. Number of unigenes related to TFs in each TF family. Among the TF families, bHLH, MYB, C3H, and WRKY proteins were the most abundant.

Temporarily and spatially regulating the activities of target genes, transcription factors (TFs) play key roles in plant development and stress response. TFs are often classified into different families according to the features of DNA-binding domains. *Glycine max *was previously found to contain 5,069 TFs from 57 families (Plant Transcription Factor Database, PlantTFDB, http://planttfdb.cbi.pku.edu.cn[29]). Subsequently sequence search using extracted *Glycine max *TFs as query (blastn program with e ≤10^-5^) was performed, and as a result, 8,025 TF candidates were identified in *A. mongolicus*. There are totally 6,359 TFs (79.23%) significantly expressed (RPKM ≥ 0.5), of which 2,676 were commonly expressed in all three tissues (Figure 6). Many different expression patterns for TFs were observed. Some TFs are unique to each tissue, whereas many others were shared by two of the three (Figure 6). Root and stem shared the most expressed TFs (1,030), followed by leaf and stem (490). On the other hand, stem has the largest number of tissue-specific TFs (839). Among the TF types, bHLH, MYB, C3H, and WRKY proteins were the most abundant, with the least (MYB) having 318 members, and the largest (bHLH) having 951 (Figure 7) (Additional file 4).

#### bHLH proteins family

**Figure 8:**
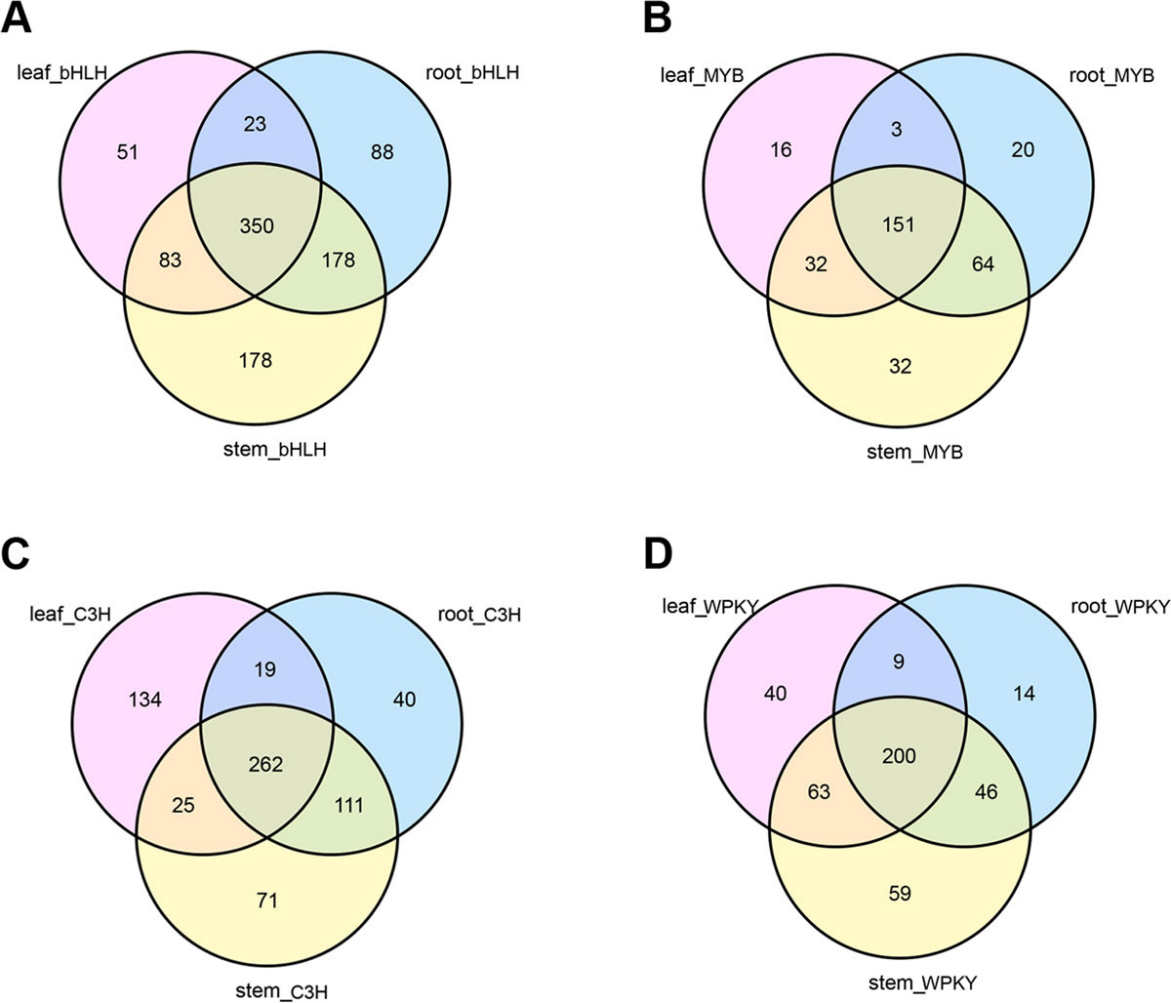
**Related unigenes of the top TFs in three tissues**. Unigenes with RPKM≥0.5 were considered as expressed in each tissue. A, Venn diagram showing expressed bHLHs in three tissues. B, Venn diagram showing expressed MYBs in three tissues. C. Venn diagram showing expressed C3Hs in three tissues D. Venn diagram showing expressed WRKYs in three tissues

The bHLH proteins are widely distributed in all three eukaryotic kingdoms and form one of the largest TF families[30]. In plants, bHLH proteins engage in the regulation of broad biological processes, including light signaling[31], hormone signaling, wound and drought stress responses [32,33], and organ tissue development[34]. In *A. mongolicus*, our analysis showed that root, stem and leaf have 639, 789 and 507 bHLH proteins expressed, respectively (Additional file 4). The expressed pattern of bHLH proteins in each tissue was shown in Figure 8A, where the stem has the largest number of tissue-specific bHLH proteins (178). Plant bHLH proteins were classified into 32 subfamiles based on genome-wild classification and the evolutionary analysis [30], whereas members of the same plant bHLH subfamiles had similar functionally characteristic [35]. There were some bHLH subfamiles involved in the control of phenylpropanoid biosynthetic pathway in higher plant, especially flavonoid /anthocyanin metabolism, such as PsGBF [36], and R gene[37]. The R gene product Lc was the first plant protein reported to possess a bHLH domain in maize [37], in addition to AtTT8 in Arabidopsis and OsRa-c in rice[38,39].

#### MYB proteins family

Some plant bHLH proteins were known to form transcription complexes with MYB proteins[40]. The first plant MYB gene, C1, was isolated from *Zea mays *and encodes a *c-myb-like *TF involved in anthocyanin biosynthesis [41]. Many functions of MYB proteins were reported, including regulation of gene expression, secondary metabolism, and response to environmental stresses[42]. There are 155, 197 and 369 MYB genes in rice, *Arabidopsis*, and soybean, respectively (PlantTFDB, http://planttfdb.cbi.pku.edu.cn/) [43]. In comparison, from our analysis, a total of 318 MYB proteins were found in *A. mongolicus*, and the number of MYB unigenes in root, stem and leaf were 239, 280 and 202, respectively (Additional file 4). There were 151 MYB unigenes commonly expressed in all three tissues (Figure 8B). MYB proteins are characterized by a highly conserved DNA-binding domain, which are divided into three classes depending on the number of adjacent repeats [44]. The MYB family possesses the most members in plants, which are involved in the regulation of secondary metabolism, including flavonoid biosynthesis. For example, *AtMYB11/PFG1, AtMYB12/PFG1 *and *AtMYB111/PFG3 *regulated flavonol biosynthesis in all tissues; *AtMYB75/ PAP1, AtMYB90/PAP2, AtMYB113 and AtMYB114 *regulated anthocyanin biosynthesis in vegetative tissues; *AtMYB123/TT2 *regulated proanthocyanidins (PAs) biosynthesis in the seed coat; and *AtMYB5 *are involved in tannin biosynthesis of *Arabidopsis*[44]. Much work remains to be done to classify the MYB genes from *A. mongolicus *and characterize their expression and functions of the subfamilies.

#### C3H Proteins family

The C3H proteins, which have C-X_n_-C-X_m_-C-X_3_-H motifs, are a large family of zinc finger proteins. Previous studies suggested that they may have RNA-binding activity in pre-mRNA processing[45]. There were a large number of tissue-specific C3H proteins revealed in the leaf (134), stem (71), and root(40) in *A. mongolicus*, respectively (Figure 8C). In contrast, 68 and 67 C3H family genes were identified in Arabidopsis and rice, respectively[46]. Many zinc finger families of proteins were found in plants, including RING-finger, ERF, WRKY and DOf family, which are involved in transcriptional regulation of growth development and tolerance ability to adversity stress[46]. In addition to C3H proteins, other members of zinc finger families were found from *A. mongolicus *in our study, including WRKY, C2H2 and ERF related genes (Figure 7).

### Analysis of secondary metabolic pathways in *A. mongolicus*

From our analysis, 13,057 unigenes were annotated with Enzyme Commission (EC) numbers and assigned to 339 reference canonical KEGG pathways, extending the previous study[25], which assigned a total of 2877 unigenes to 45 KEGG pathways. The extended gene and pathway lists probably resulted from the multiple tissue samples used in our current study, which is complimentary to the early work in term of both sequencing technology platform and amount of tissue data generated. Besides the conservative primary metabolic pathways, we observed some great expansions in the enzyme families involved in the secondary metabolic pathways. We will next focus our analysis on the isoflavonoids biosynthesis and triterpene saponin biosynthesis pathways, due to their close association with the pharmacological activities of *A. mongolicus*.

#### Isoflavonoids biosynthesis

##### Isoflavonoids biosynthetic pathway

**Figure 9:**
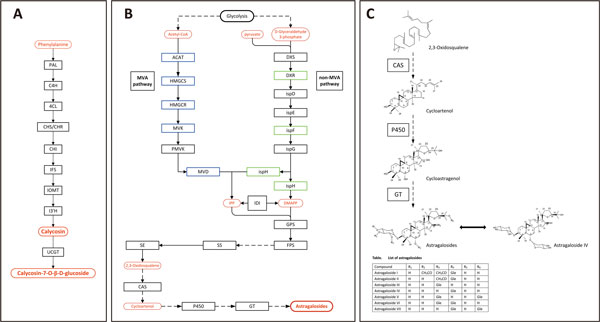
**Two secondary metabolic pathways in *A. mongolicus***. A) Enzymes involved in the pathway of isoflavonoid biosynthesis. B) Enzymes involved in the putative pathway of triterpenoid biosynthesis. Blue boxes indicate the highly expressed enzymes in root and stem, while green boxes indicate the highly expressed in leaf. Red boxes indicate important compounds in the pathway. C) Structural formulas of compounds synthesized in the putative downstream pathway of astragalosides biosynthesis.

Isoflavonoids are a subclass of flavonoids with unique structure and function produced mainly in legumes. They possess features of antibacterial and antioxidant, which improve the survival of leguminous plants in nature. Isoflavonoids also possess a wide range of clinical activities, including prevention of bone loss, osteoporosis, cancer, and neurodegenerative disease[47]. In addition, isoflavonoids are a type of phytoestrogen with a molecular weight and structure similar to estrogen, which could complement the estrogen inside body, and alleviate estrogen-related diseases through two-way regulation[48]. As a traditional herb, the bioactivities of *A. mongolicus *are closely related to its isoflavonoids, including formononetin, calycosin and CG, which are synthesized via the isoflavonoids pathway(Figure 9A) [9].

The isoflavonoids pathway in *A. mongolicus *was characterized based on other legumes[10,11]. Calycosin and CG are synthesized from L-phenylalanine via the isoflavonoids branch of phenylpropanoid metabolism (Figure 9A). It starts with Phenylalanine ammonia lyase (PAL), which catalyzes the deamination of L-phenylalanine to form cinnamic acid[49]. Cinnamate-4-hydroxylase (C4H) adds a hydroxyl group to produce 4-Coumarate, and then links CoA by 4-coumarate coenzyme A ligase (4CL)[50]. Chalcone synthase (CHS) catalyzes the key condensation of 4-coumaroyl CoA and three molecules of malonyl CoA, and co-acts with chalcone reductase (CHR) to synthesize isoliquritigenin, which is then converted to 7,4'-Dihydroxyflavanone by chalcone isomerase (CHI)[51]. Isoflavone synthase (IFS) converts flavanones to their corresponding isoflavones with a 2, 3 aryl ring migration. Downstream formononetin is formed under isoflavone 4'-O-methyltransferase (IOMT). Subsequently, isoflavone 3'-hydroxylase (I3'H) catalyzes the hydroxylation reaction at the 3'-position to generate calycosin, which is transformed into CG under calycosin 7-O-glucosyltransferase (UCGT)[52].

In previous studies, we cloned cDNA sequence of most genes involved in the isoflavonoids biosynthesis from *A. mongolicus*. Some of them included multigene families (*PAL *and *CHS*)[9]. Complete cDNA sequence encoding *CHR *[GenBank: HM357239], *CHI *[GenBank: DQ205407], and partial cDNA sequence of two *PAL *genes [GenBank: EF110924 and EF110925], four *CHS *genes [GenBank: DQ140415, EF110921, EF110922, and EF110923], and *C4H *[GenBank: DQ371297] had been cloned and submitted in NCBI database. In addition, we cloned complete cDNA of *IFS *[GenBank: JN828952] and *I3'H *[GenBank: JQ609280], and modified gene sequence to increase protein solubility for successful expression and characterization (unpublished data).

##### Tissue-differential expression of involved genes

**Figure 10:**
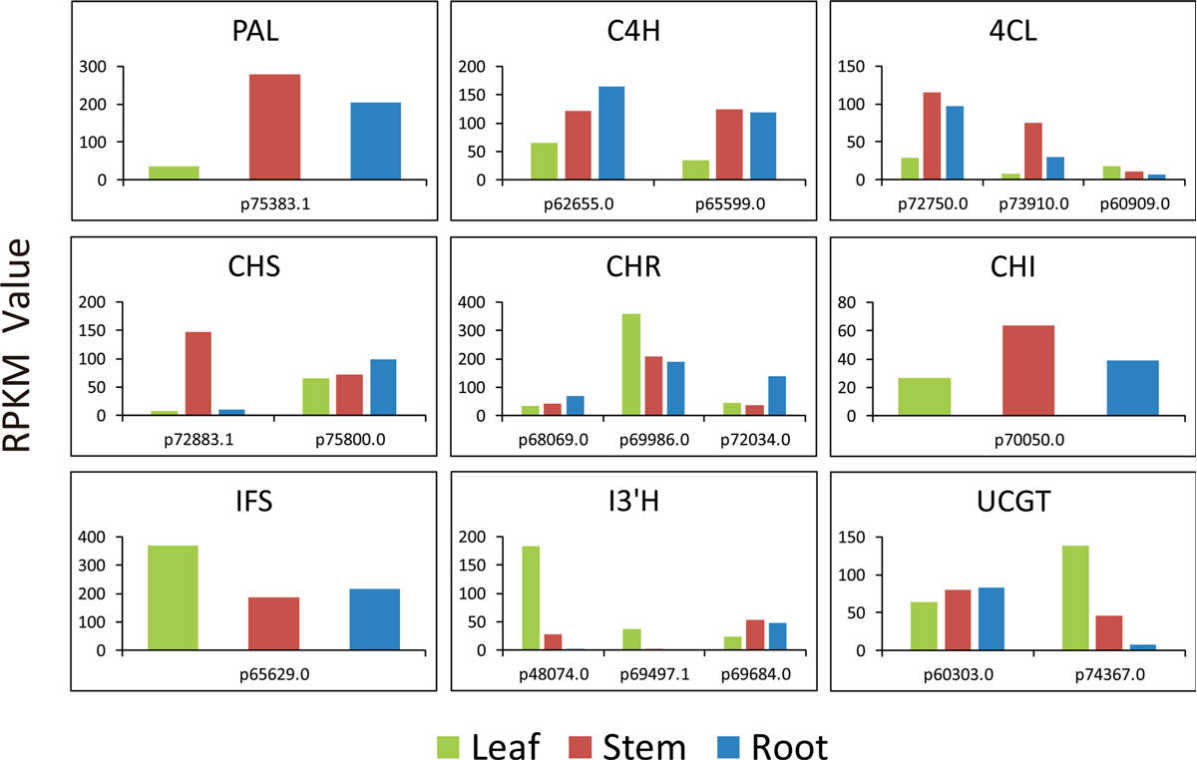
**Expression level of genes involved in isoflavonoids biosynthesis in three tissues**. Nine genes involved in the isoflavonoids biosynthesis showing different expression patterns.

In this study, we investigated the expression patterns of genes involved in the biosynthesis of calycosin and CG in different tissues of *A. mongolicus*. All predicted enzymes involved in the isoflavonoids biosynthesis were discovered in *A. mongolicus *transcriptome dataset except IOMT, and most genes were found with several isoforms (Figure 10). The transcriptional level of most genes were lowest in the leaves, and the highest in the stem. *PAL *(p75383.1) had the highest expression in the stem, followed by the root, and the leaf. The expression patterns of one *C4H *isoform (p65599.0), *CHS *(p72883.1), *I3'H *(p69684.0), two *4CL *isoforms (p72750.0, p73910.0), and *CHI *(p70050.0) were similar to that of *PAL*. Other isoforms of *C4H *(p62655.0), *CHS *(p75800.0), *UCGT *(p60303.0) and *CHR *(p68069.0, p72034.0) displayed different expression pattern, with highest level in the root, followed by the stem and the leaf. *IFS *(p65629.0), one *4CL *isoform (p60909.0), *CHR *(p69986.0), *UCGT *(p74367.0) and *I3'H *(p48074.0, p69497.1) were different with the highest expression level in the leaf, followed by the stem and the root (Figure 10).

It's interesting to find that isoforms of genes display distinctive expression patterns. Both *C4H *and *CHS *had two isoforms, and each of the isoforms showed different expression pattern. This phenomenon was more obvious with three isoforms of *I3'H*, for which two of them (p48074.0, p69497.1) showed the highest expression in the leaf, and whereas for the third (p69684.0) its expression was the lowest in the leaf (Figure 10). The biological significance of the distinctive expression patterns for isoforms remains to be the focus of future study.

Unlike the genes in the isoflavonoids biosynthetic pathway, *I3'H *isoforms (p48074.0, p69497.1) and *UCGT *(p74367.0), the enzymes that synthesize calycosin and CG, exhibited significantly higher expression level in the leaf compared to other tissues. The expression levels of *I3'H *(p48074.0, p69497.1) and *UCGT*(p74367.0) in the leaf were 6-, 13-, and 3-fold higher compare to those in stem, respectively; and were 64-, 55-, and 18- fold higher compared to those in root. For the other *I3'H *isoform (p69684.0) and *UCGT *(p60303.0), their expression levels didn't have much difference among three tissues. This result was in accordance with the previous study[9], in which Pan et al. used QRT-PCR analysis to investigate the expression patterns of several genes in leaf, stem and root from *A. mongolicus. I3'H *and *UCGT *both had the highest expression in the leaf among three tissues. Pan et al. also reported none calycosin content was detected in leaves and stems, but both calycosin and CG were abundant in the root. Considering natural compounds are often transported from synthetic site to the site of accumulation in plants[53], we propose that calycosin and CG may be synthesized mainly in the leaves, and were transported to roots for storage.

Previous studies used quantitative real-time PCR to investigate the gene expression related to the biosynthesis of isoflavonoids in different *A. mongolicus*[9,54]. Four of five related genes expressed the highest in the leaf at 16°C(*CHS, IFS, I3'H, UCGT*), except *PAL*[9]. For the nine genes including *PAL, C4H, 4CL, CHS, CHR, CHI, IFS, I3'H, UCGT *at 25°C in another study, *I3'H *was highly expressed in the root compared to other tissues, while *I3'H *and *UCGT *showed lowest expression level in the leaf[54]. However, the two earlier studies failed to detect different expression pattern of gene isoforms, whereas our study was the first to identify many isoforms of the isoflavonoids biosynthetic genes, and observed their different expression patterns among tissues. Provided the different growth environments and culture conditions, it is likely that the biosynthesis pathway of isoflavonoids in *A. mongolicus *is influenced by the stage of growth cycle and environmental conditions. Our results may pave a way to further understand the regulation mechanisms of isoflavonoids biosynthesis in different tissues.

#### Triterpenoid saponins biosynthesis

##### Distinct pathways for terpenoid precursors biosynthesis in different tissues

In higher plants, the biosynthesis of triterpenoid saponins, which is a highly complex metabolic network, generates a wide range of molecular structures. Its biosynthetic pathway could be generally divided into three stages. In the Initial stage, the universal terpenoid precursors, isopentenyl diphosphate (IPP) and dimethylallyl diphosphate (DMAPP) are synthesized. By labeling using [1-^13^C] glucose, two distinct biosynthetic routes were found for the synthesis of IPP. One is the known mevalonate (MVA) pathway, which exists mainly in the cytoplasm and mitochondria, and mainly synthesizes sterols, ubiquinones and sesquiterpenes[55]. The other is the non-MVA (MEP/DOXP) pathway, which operates inside the plastid compartment, and produces polyterpenes along with the chloroplast-bound isoprenoids (β-carotene, lutein, prenyl chains of chlorophylls and plastoquinone) [56]. Downstream product of IPP, geranyl pyrophosphate (GPP) is formed under geranylgeranyl diphosphate synthase (GPS). And then farnesyl diphosphate synthase (FPS) catalyzes the conversion from GPP to farnesyl pyrophosphate (FPP). In the stage of carbon skeleton formation, the biosynthesis of suqalene is catalyzed by squalene synthase (SS), and then suqalene is converted into 2,3-oxidosqualene by squalene epoxidase (SE). Finally, the cyclization of 2,3-oxidosqalene is catalyzed by oxidosqualene cyclase (OSC) that generates triterpenoid skeleton. In the modification stage, little is known about the downstream biosynthetic pathway after cyclization in saponins biosynthesis. Some cytochrome P450 monooxygenases (CYP450s) and glycosyl transferases(GTs) are assumed to do modifications on triterpenoid skeletons, including hydroxylation and glycosidation, which leads to the generation of various triterpenoids (Figure 9B). All predicted enzymes involved in the triterpene saponin biosynthesis were discovered in the *A. mongolicus *transcriptome dataset.

**Figure 11:**
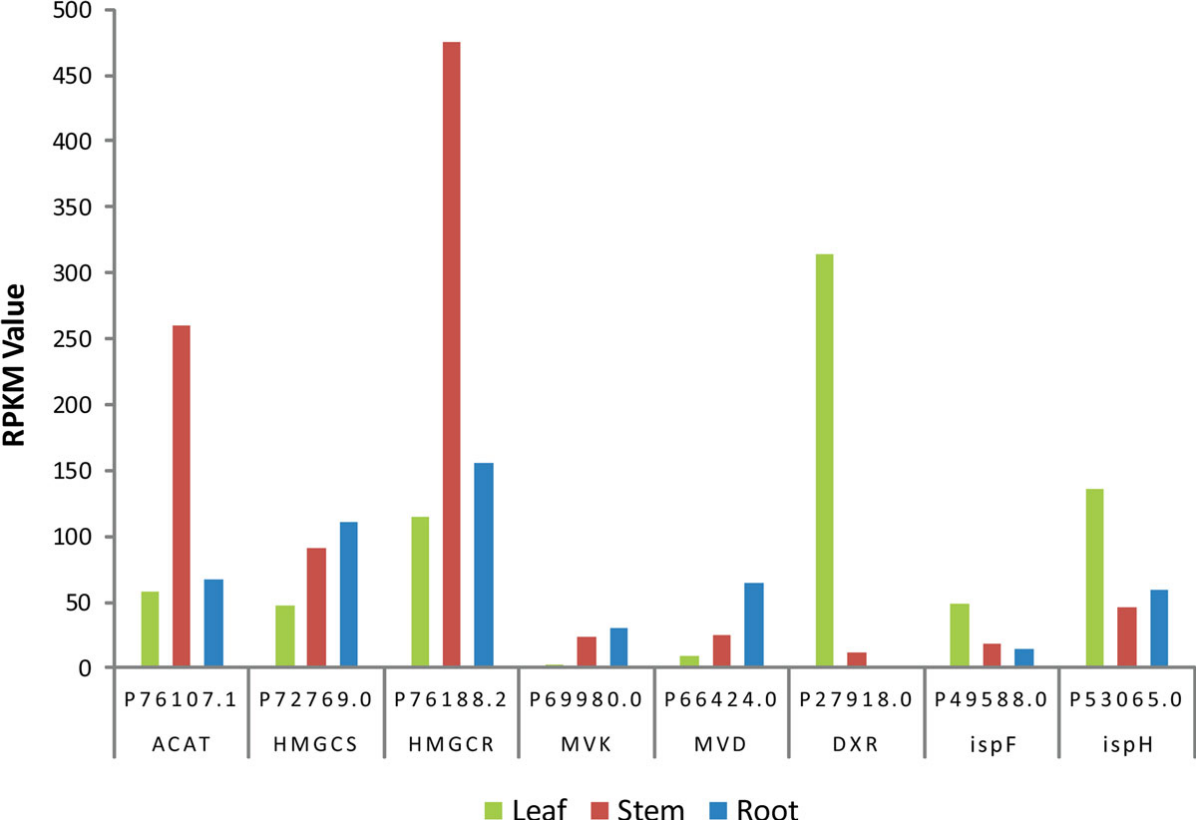
**Tissue-differential expressed genes involved in triterpenoid saponins biosynthesis**. *ACAT, HMGCS, HMGCR, MVK *and *MVD *from MVA pathway were highly expressed in the root and stem. *DXR, ispF *and *ispH *from non-MVA pathway were highly expressed in the leaf.

As we mentioned before, IPP and DMAPP biosynthesis follows two distinct pathways in higher plants. The conventional mevalonate (MVA) pathway is used in cytoplasm, whereas the non-mevalonate (MEP/DOXP) pathway operates in chloroplasts[56,57]. It is striking that the eight genes in MVA and non-MVA pathways are found to be differentially expressed among the three tissues (Figure 11). In contrast to that in the leaf, acetyl-CoA C-acetyltransferase (*ACAT*), hydroxymethylglutaryl-CoA synthase (*HMGCS*), hydroxymethylglutaryl-CoA reductase (*HMGCR*), mevalonate kinase (*MVK*) and diphosphomevalonate decarboxylase (*MVD*) from MVA pathway were highly expressed in the root and stem. 1-deoxy-D-xylulose-5-phosphate reductoisomerase (*DXR*), 2-C-methyl-D-erythritol 2, 4-cyclodiphosphate synthase (*ispF*) and 4-hydroxy-3-methylbut-2-enyl diphosphate reductase (*ispH*) from non-MVA pathway, however, were highly expressed in the leaf (Figure 11). So it clearly indicates that non-MVA pathway was the choice for the leaf, in which chloroplasts are abundant, whereas MVA pathway is preferred in the root and stem. The results of differentially expressed genes in the two pathways among tissues, strongly suggest the existence and the regulation of the parallel but compartmentally separated biosynthesis pathways of IPP and DMAPP in the difference tissues of *A. mongolicus*.

##### Discovery of an OSC gene, CAS, from A. mongolicus

Plants possess structurally diverse saponins that are subdivided into 11 main classes based on their carbon skeletons, such as dammaranes, lupanes, taraxasteranes, ursanes, cycloartanes, steroids and so on[58]. To form these skeletons, plants encode multiple OSCs including lupeol synthase (LS), dammarenediol-II synthase (DS), β-amyrin (β-AS) and cycloartenol synthase (CAS), which have been cloned in plants[13]. The classification and synthetic pathway of ginsenosides from *Panax *species have been researched, and more than 150 naturally ginsenosides have been isolated and identified. Most of them belong to dammarane-type, whereas others are oleanane-type[59]. So far, at least twenty genes related to the biosynthesis of ginsenosides have been functional identified, including two OSC genes. DS catalyzes the cyclization of 2,3-oxidosqualene to form the dammarane skeletons, and β-AS participates in the formation of oleanane-type ginsenosides[13,60]. In comparision with *Panax *species, less than 20 triterpenoid saponins were isolated in *A. mongolicus *till now, and most of them are classified as cycloartane-type triterpenoids[61]. Studies about the rate-limited step in triterpenoid saponins biosynthesis, the cyclization catalyzed by OSCs, are also limited.

In this study, we detected the existence of a high-quality *CAS *transcript (p75841.1), and no other OSC genes were found. To experimentally validate the *CAS *transcript obtained from RNA-seq sequencing, we cloned the *CAS *gene from *A. mongolicus *(*AmCAS*). The *AmCAS *sequence was more than 2000 bp, and thus divided into two fragments for clone and sequencing. Both fragments were PCR positive at the expected size and confirmed by sequencing (The ORF sequence (2274 bp) of *AmCAS *and primers designed for PCR analysis were shown in additional file 5).

**Figure 12:**
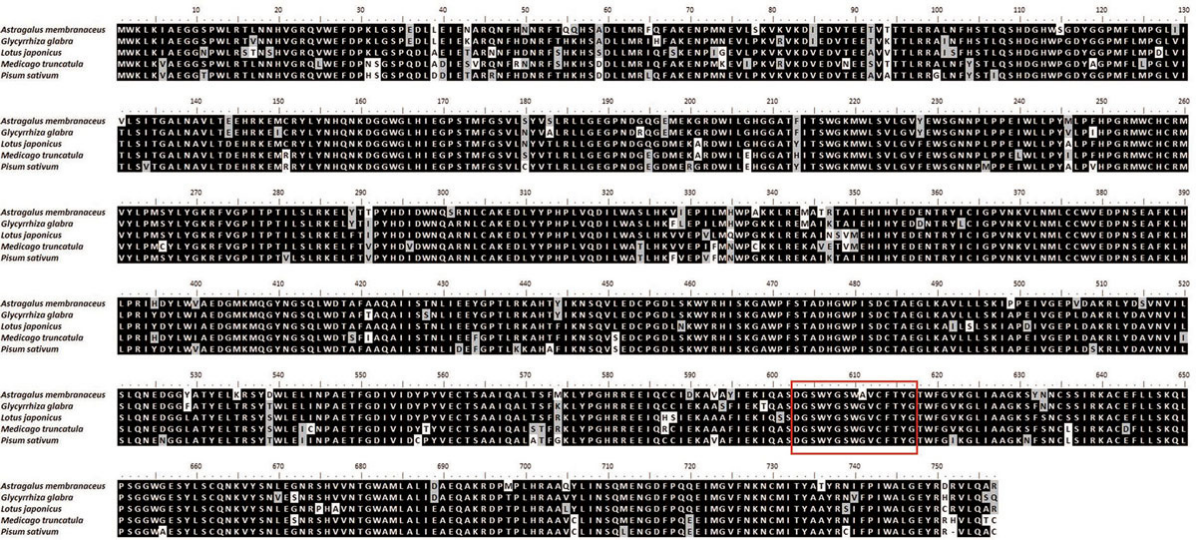
**Protein sequence alignment of CAS from *A. mongolicus, Glycyrrhiza glabra, Lotus japonicas, Medicago truncatula *and *Pisum sativum***. AmCAS and *Glycyrrhiza glabra *CAS showing 93% identity, AmCAS and *Lotus japonicas *CAS showing 90% identity, AmCAS and CAS from *Medicago truncatula *and *Pisum sativum *showing 88% identity. Red box indicates the terpene synthases signature.

With the plant *CAS *genes having been cloned and characterized from a gymnosperm, several monocots and numerous edudicots[62], we performed the homology analysis with NCBI Blast tool (http://blast.ncbi.nlm.nih.gov/Blast.cgi). The results showed that AmCAS is 93% identical to CAS from *Glycyrrhiza glabra *[GenBank: Q9SXV6.1], 90% identical to CAS from *Lotus japonicus *[GenBank: BAE53431.1] and 88% identical to CAS from both *Medicago truncatula *[GenBank: XP_003610947.1] and *Pisum sativum *[GenBank: BAA23533.1]. The amino acid sequence of AmCAS was aligned with above four plants (Figure 12). We also scanned our protein sequence against the PROSITE collection of motifs (http://prosite.expasy.org/), and identified a perfect terpene synthases signature: [DE]-G-S-W-x-[GE]-x-W-[GA]-[LIVM]-x-[FY]-x-Y-[GA] ( Figure 12) [63]. The similarity of *AmCAS *genes to those in other plants indicates that they are orthologous[17]. We proposed that AmCAS catalyzes the cyclization of 2,3-oxidosqualene to form cycloartenol and further generate ASI in *A. mongolicus *(Figure 9B &8C). In addition to ASI, there are multiple astragalosides that share common carbon skeleton with several different substituents. We suggested AmCAS also play an important role in the biosynthesis of these astragalosides. This study provides a good foundation for further researches on the biosynthesis of astragalosides.

##### Discovery of the CYP450s and GTs involved in triterpene saponin biosynthesis by phylogenetic and co-expression analysis

In contrast to the chair-chair-chair conformation during synthesis of most triterpenoid saponins catalyzed by OSCs such as DS and β-AS, CAS converts 2,3-oxidosqualene backbone into a chair-boat-chair conformation[17]. Then, several specific CYP450s might catalyze cycloartenol into cycloastragenol. Subsequently, glycosyltransferases (GTs) transfer the activated saccharides to an aglycone substrate, which involved in the conversion of cycloastragenol to numerous astragalosides. With the comparision of molecular structure between ASI and cycloartenol, it is obvious that ASI has four more hydroxyls at C-6, -16, -24 and -25 separately, and two more glycosyls. Based on these differences, we propose there are at least 4 CYP450s and 2GTs catalyze the following modification on cycloartenol (Figure 9C). The CYP450s constitute an important superfamily of highly divergent sequences that could be divided into 10 clans. Among these clans, CYP72 clan are involved in the catabolism of isoprenoid hormones; CYP71 clan modify the shikimate products and intermediates; CYP85 clan participate in the modification of cyclic terpenes and sterols in the brassinosteroid pathway[64]. And further studies have identified CYP93E1 from *Glycine max *(CYP71 clan) [65] and CYP88D6 from *Glycyrrhiza uralensis *(CYP85 clan) [66], both of which were involved in saponin biosynthesis. Thus, the CYP71, CYP72, CYP85 clan might be involved in the astragalosides biosynthesis in *A. mongolicus*. Our assembly sequences contained 95 unigenes annotated as CYP450s, of which 53 were classified as CYP71 clan, 4 as CYP72, and 21 as CYP85 (Additional file 6).

As reported from study on *Medicago truncatula *and *Panax ginseng*, enzymes in the same biosynthetic pathway are usually co-expressed[15,67]. Hence, in order to further narrow the potential range of CYP450s and GTs that involve in the biosynthesis downstream of astragalosides, we performed a co-expression analysis with CAS and its upstream enzyme, squalene expoxidase (SE). Co-expression between individual transcripts was assessed using pearson correlation coefficients (PCC) between RPKM values across all three tissues. Both two isoforms of *SE *(p72959.0, p70052.1) showed high co-expression (r>0.96) with *CAS *(p75841.1). This result indicated that downstream *CYP450*s and *GT*s may be similarly co-expressed with *CAS*. Co-expression analysis between all *CYP450*s and *CAS *transcripts identified 22 candidate *CYP450*s which highly co-expressed with *CAS *(r>0.9) across three tissues. There were 7 transcripts belong to CYP71 clan (p57867.0, p5215.0, p7366.0, p50274.0, p26377.0, p71378.0, p60800.0), 1 transcripts belong to CYP72 clan (p71249.0), 9 transcripts belong to CYP85 clan (p52746.0, p50482.0, p60763.0, p51105.0, p65633.0, p32417.0, p69412.0, p69412.2, p43338.0). Likewise, our assembly sequences contained 92 unigene annotated as *GT*s, of which 25 *GT *unigenes were highly co-expressed with the putative *CAS *(r>0.9, Additional file 7).

##### Summary

Previous studies demonstrated that astragalosides have rich pharmacological activities. ASI as its representative compound, could exhibit anti-inflammatory function by inhibiting NF-kap-paB pathway[19], increase antibody production, T, B lymphocyte proliferation and fibrinolytic potential of cultured human umbilical vein endothelial cells[68]. Clinical experiments proved that ASI could alleviate the symptoms of congestive heart failure by improving the left ventricular modeling and ejection function[69]. ASI could also improving cerebral and myocardial ischemia et al by scavenging the active oxygen free radicals[70,71]. In spite of the important medicinal value of astragalosides, the mechanism and involved enzymes of downstream biosynthesis of astragalosides is little known at present[12]. In this study, we identified a key enzyme, CAS, several candidate CYP450s and GTs involved in downstream biosynthesis of astragalosides. These results will pave the way to functional characterized these genes and further clarify the biosynthesis pathway of astragalosides and other triterpene saponins in *A. mongolicus*.

###### Analysis of CYP450s in *A. mongolicus*

CYP450 enzymes are a class of protein superfamily that catalyze the oxidation of organic substances, which are widely distributed in animals, plants, fungi and bacteria. There are 244 *CYP450 *genes in *Arabidopsis *genome and more than 1000 *CYP450 *isoforms expected in wheat, thus *CYP450*s constitute one of the largest gene families in plants[72]. The *CYP450*s sometimes share less than 20% identity, which leads to limited functional redundancy. They catalyze diverse reactions, including the bioconversion of drugs, terpenes, alkanes and aromatic compounds, the biotransformation of xenobiotics, the metabolism of chemical carcinogens, and the biosynthesis of important biological compounds, such as saponins, flavonoids, fatty acids, fat-soluble vitamins, steroids and bile acids[72,73]. The family and function diversification of CYP450s reflect the complexity of plant metabolism, thus great attention was paid to CYP450s in *A. mongolicus*. It is difficult to accurate annotate and predict molecule function of *CYP450 *genes with high sequence identity, thus 95 predicted unigenes contained in our assembly were annotated as 138 *CYP450*s (Additional file 6). We performed phylogenetic analysis with these *CYP450*s, compared the diversity of CYP450 clans among *A. mongolicus, Medicago truncatula *and *Arabidopsis thaliana*, and further analyzed tissue specific expression of these *CYP450*s.

**Figure 13:**
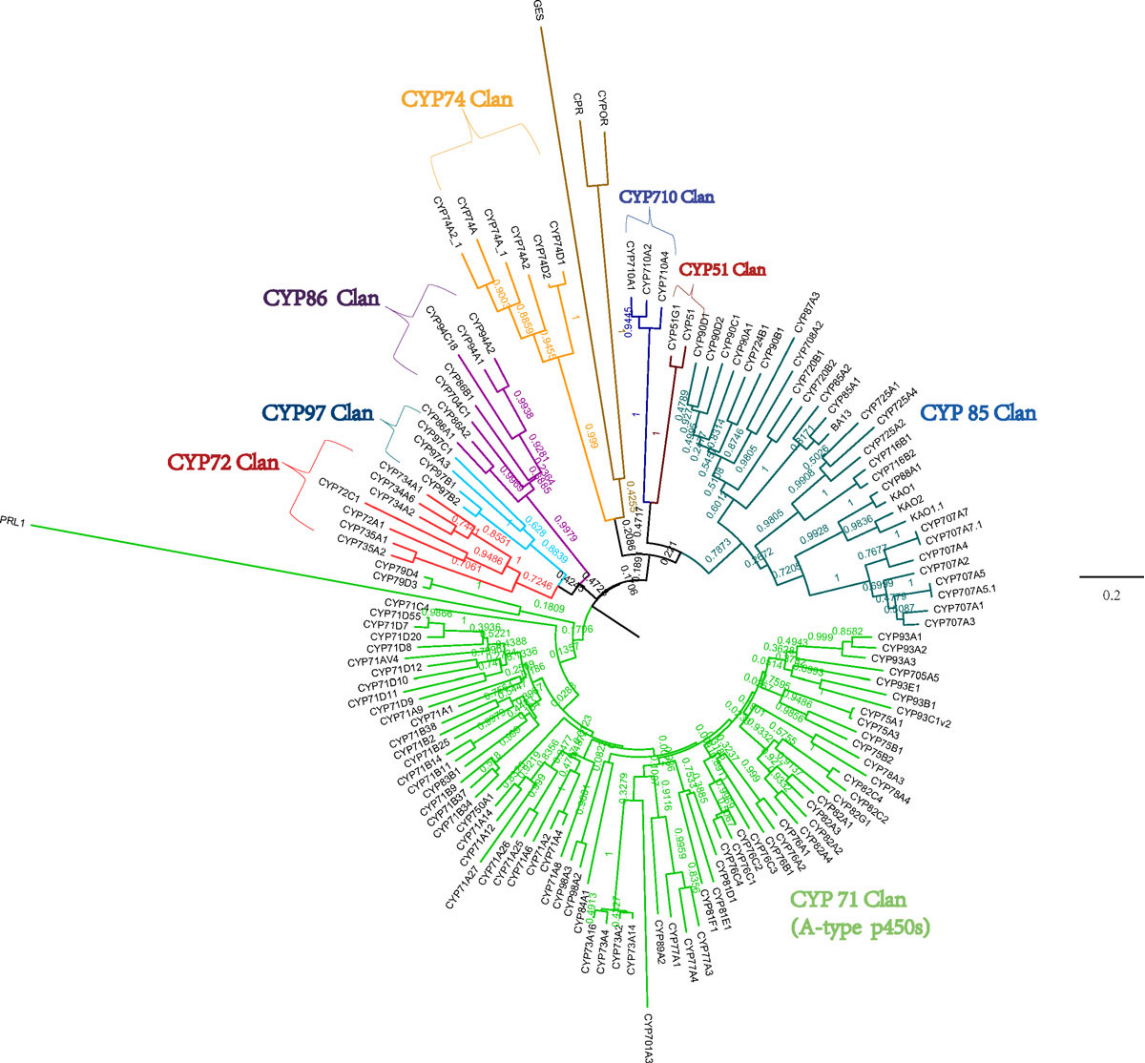
**The neighbor-joining bootstrapped phylogenetic tree of *A. mongolicus *P450 genes**. Bootstrap values which have been converted into the percentage obtained after 1000 replications are indicated on the branches.

A neighbor-joining bootstrapped phylogenetic tree was built based on multiple alignments of CYP450 amino acid sequences to analyze their evolutionary relationship (Figure 13). A total of 135 sequences were sorted into 8 clans according to previous study[64]. Four clans, CYP51, CYP74, CYP97 and CYP710, are single family. The other four, CYP71, CYP72, CYP85 and CYP86, contain multiple CYP450 families in each. With 135 Cytochrome C Oxidases divided into the 8 clans, the rest three P450s (GES, CPR and CYPOR) were found to belong to Cytochrome C Reductase.

Single family CYP450 clans usually have ancestral functions such as sterol, lipid and carotenoid metabolism and signaling, whereas multiple family CYP450 clans have evolved by intensive gene duplication and diversification with a major evolutionary burst in Angiosperms[74]. CYP51 clan is one of the oldest and the most conserved eukaryotic CYPs. CYP710s act following CYP51s in sterol biosynthesis. Only two unigenes annotated as CYP51G1, CYP51G2P separately exist in *A. mongolicus*, which could be considered as the prototype of a stable and highly conserved CYP due to high selection pressure[72]. CYP74s are untypical CYP450s that catalyze reaction without electron donors and molecular oxygen. They synthesize allene oxide in the jasmonate, and act as divinyl ether synthase and hydroperoxide lyase to generate antimicrobial or signaling compounds[75,76]. CYP97 clan are all plastidial enzymes that catalyze hydroxylation of carotenoids to form essential components of the light-harvesting systems[77]. The multiple-family clans, especially CYP71, CYP72, CYP85, have expanded greatly. It is remarkable that CYP71 clan is much bigger than the rest clans. In fact, CYP71 clan account for more than 50% of plant CYP450s with a big diversity of function, such as modifications of shikimate products and intermediates, metabolism of small isoprenoids, some triterpenoid derivatives, fatty acids, aromatic and aliphatic amino acid derivatives, and so on[74]. CYP72 clan is involved in the catabolism of isoprenoid hormones and the biosynthesis of cytokinins[74,78]. CYP85 clan is devoted to the modification of sterols and cyclic terpenes in the brassinosteroid, abscisic acid, and GA pathways[79]. Contrasting with other three clans, CYP86 is a more conservative clan with only four families. CYP86s exclusively catalyze hydroxylation of fatty acids, fatty alcohols or alkanes and their derivatives[80].

**Figure 14:**
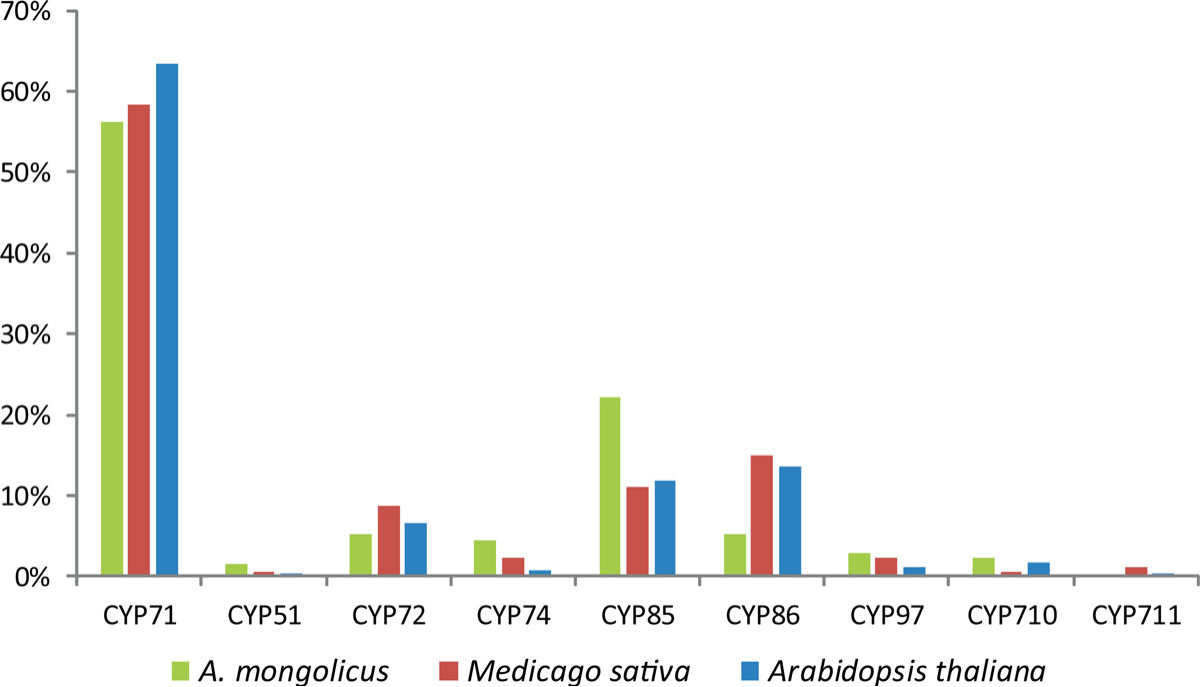
**Proportion of each CYP450 clan of *A. mongolicus, Medicago truncatula *and *Arabidopsis thaliana***. Proportion of nine CYP450 clans of three species were compared, respectively. The distribution trend of CYP450 clans is basically consistent in all three species.

To compare the similarities and differences exist in CYP450 clans between *A. mongolicus *and other species, we summarized the sequences number and proportion of different CYP450 clans of *A. mongolicus, Medicago truncatula *and *Arabidopsis thaliana *(Additional file 6, Figure 14). The distribution trend of CYP450 clans is basically consistent in all three species. It's also worth noting that the proportion of CYP85 clan in *A. mongolicus *is almost twice as large as in *Medicago truncatula *and *Arabidopsis thaliana *(Figure 14). This agrees with the fact that terpenes is one of main secondary metabolites in *A. mongolicus *that is modified by CYP85 clans.

**Figure 15:**
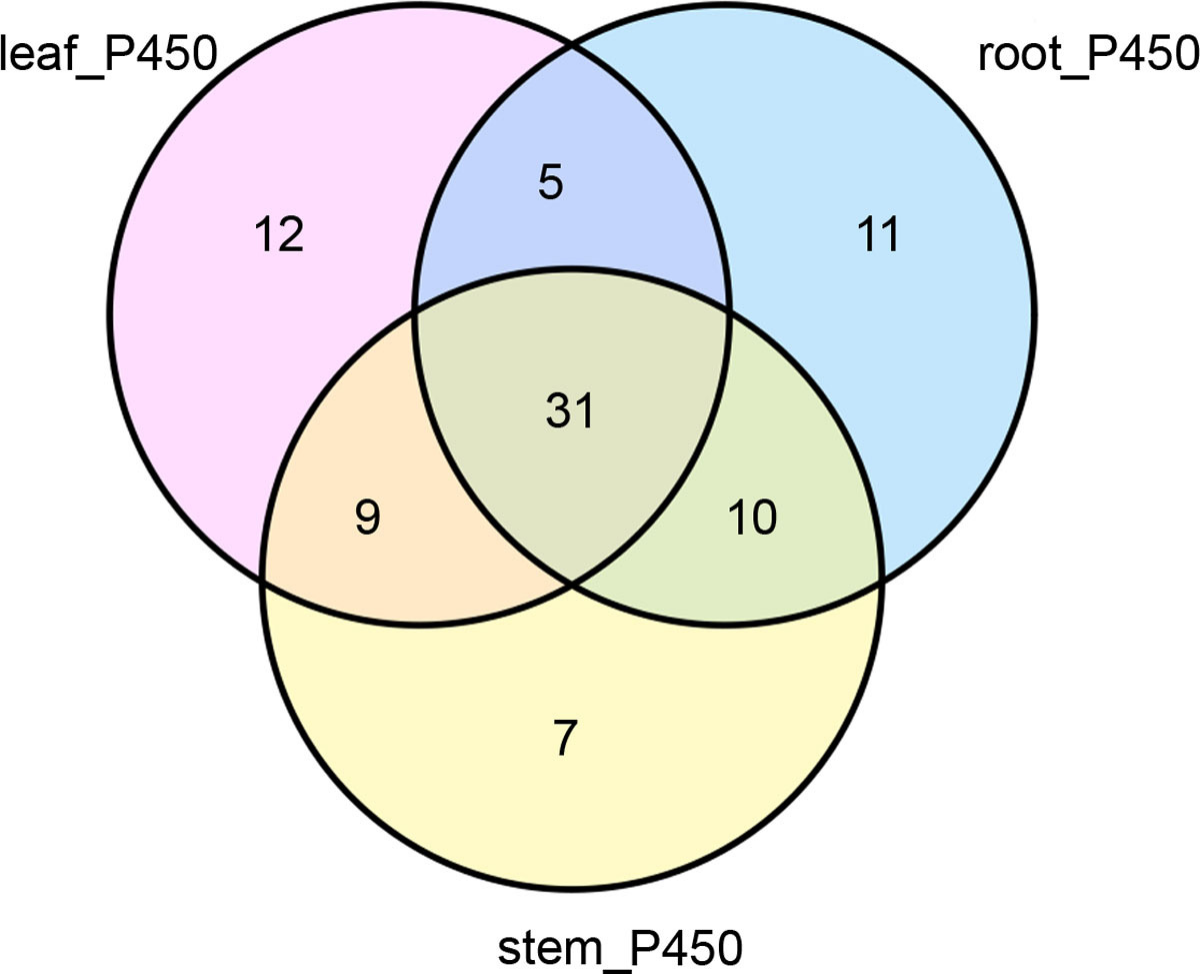
**Venn diagram showing expressed CYP450s in three tissues**. A total of 85 CYP450s (89.47%) are expressed in three tissues, of which 31 CYP450s were commonly expressed in all three tissues. Each tissue had 57 expressed CYP450 unigenes. Unigenes with RPKM≥0.5 were considered as expressed in each tissue.

To investigate the tissue specific distribution of CYP450s, we compared the number of unigenes annotated as CYP450 expressed in each tissue (with RPKM≥0.5 as a cut-off). A total of 85 *CYP450*s (89.47%) were expressed in three tissues, and out of them, 31 were commonly shared in all three tissues (Figure 15). Each tissue had 57 expressed *CYP450 *unigenes. Leaf expressed the most tissue-specific CYP450 (12), followed by the root (11) and stem (7). The tissue specific distribution of CYP450s could be due to the partition of the metabolic pathways and molecular functions among tissues. The information about commonly shared and tissue-specific CYP450s in three tissues was collected in Additional file 6. Our work provides an extensive analysis of CYP450s and CYP450 clans, comparing those of *A. mongolicus *with *Medicago truncatula *and *Arabidopsis thalian*. And investigated the commonly shared and tissue-specific CYP450s in three tissues. We established the diversity and phylegenetic relationships among the CYP450 families, which builds a foundation for further functional study of CYP450s in *A. mongolicus*.

## Conclusion

*A. mongolicus *is a traditional Chinese herb that has been widely used for thousands of years, but with limited genomic information. Using RNA-seq technology, a total of 159.5 million raw reads (approximate 15.9Gbp) were obtained, and assembled into 186,324 unigenes, in which 129,966 were annotated. The data provided a comprehensive coverage of *A. mongolicus *transcriptome to construct and profile their metabolic pathways in tissues, especially for those important bioactive molecules. The results revealed a comprehensive profile for metabolic activities among tissues, pointing to the equal importance of leaf and stem in the production of bioactive compounds. Further studies are needed to elucidate the regulation mechanisms in different tissues for their differential regulation of secondary metabolic pathways. This work provides valuable resources for bioengineering and in vitro synthesis of the natural compounds for medical research and for potential drug development.

## Materials and methods

### Plant materials and treatments

*A. mongolicus *were grown for 100 days at 25°C under a 16 h light/8 h dark photoperiod regime in pots that contained a mixture of vermiculite, blackland and perlite (9:3:0.5). The plants were irrigated with water and fertilized once a week. The 100-d-old plant was separated into root, stem and leave. All the tissues were immediately frozen in liquid nitrogen and stored at -80°C before RNA extraction [38].

### RNA Isolation, cDNA library construction and transcriptome sequencing

Total RNA was extracted from with RNAprep Pure Plant Kit (Tiangen, Code: DP432) from three different tissues of *A. mongolicus*, including leaf, stem and root. The quality of the isolated RNA was confirmed by 1% (w/v) agarose gel electrophoresis and RNA purity was detected by NanoDrop 2000 (Thermo Fisher, America). RNA concentration was measured using Qubit^® ^2.0 Fluorometer. A total amount of 10 ug RNA per sample was used as input material for the RNA sample preparations. The products were purified using RNeasy Micro kit (Cat#74004, QIAGEN) and enriched with PCR for preparing the sequencing library. The cDNA library was detected by Agilent 2100 Bioanalyzer. The cDNA library was sequenced from both of 5' and 3' ends on the Illummina HiSeq 2500 platform according to the manufacturer's instructions and 100 bp paired-end reads were generated.

### De novo assembly and mapping of sequencing reads and analysis

Before assembly, the raw reads of fastq format were filtered to obtain the high-quality clean reads by removing adaptor sequences, duplication sequences, ambiguous nucleotides (N) and low quality bases. Hence, raw reads containing larger than 5% 'N' rate (N ≥ 5%, N represented ambiguous bases in reads), or low quality bases (more than 20% nucleotides with quality value ≤ 10) were removed. De novo assembly were performed by Trinity (release 20130225) [26], with default parameters except parameters for CPU and memory. As a result, 186,324 unigenes with an average length of 895.05bp were obtained after assembly. The statistics of the length of the unigenes was performed by PERL program.

Bowtie2 (version 2.1.0) were used to align preprocessed reads(clean reads) back to transcripts, in order to get assembly statistics. RPKM was used to quantify expression abundance of transcripts in each sample. RPKM was defined as Reads Per Kilobase of exon model per Million mapped reads, calculating as following:

$$RPKM=\frac{total\;exon\;reads}{mapped\;reads\left(millinos\right)\;*\;exon\;length\left(Kb\right)}$$

The computation was done with a PERL script [24]. In each tissue, only the unigenes with a RPKM ≥ 0.5 were considered as expressed. Data compilation and calculation was performed by PERL program, and all of the figures were drawn by R or SigmaPlot (version12).

### Function annotation of unigenes

All assembled unigenes were searched against the Swiss-Prot, TREMBL, COG databases by blastx algorithms with a significant threshold of e ≤ 10^-5^, respectively. Rpstblastn program was used to search against CDD database, and the e-value was set to 10^-2^. PfamA annotation was carried out against the Pfam Database with default parameters. Transcription Factors of Glycine max was extracted from Plant Transcription Factor Database (PlantTFDB), and unigenes were mapped to them using blastn program with e ≤ 10^-5^. Pathway analysis was carried out using KAAS (KEGG Automatic Annotation Server, http://www.genome.jp/tools/kaas), Enzyme commission (EC) numbers were assigned to unique sequences that had BLASTX scores with cutoff values of e ≤ 10^-5^. And the Enzyme Commission (EC) numbers in the pathways mapped by unigenes were marked by green.

### CYP450 phylogenetic analysis

CYP450 amino acid sequences were aligned using the CLUSTAL W program and evolutionary distances were computed using the Poisson correction method, and a Neighbor-Joining (NJ) tree was constructed with MEGA6. Bootstrap values which have been converted into the percentage obtained after 1000 replications are given on the branches.

### Cloning of *AmCAS *ORF

Two pairs of primers, CASF1-CASR1 and CASF2-CASR2 were designed based on the assembled sequence, in order to perform the PCR amplification of *AmCAS *ORF (Additional file 5). The PCR conditions were 94°C for 1 min, 35 cycles at 94°C for 30 s, 56°C for 30 s, 72°C for 2 min and, finally, 72°C for 10 min. The PCR specific products were sequenced and then compared with the assembled sequence for *AmCAS *using the clustalx program.

## Abbreviations

*A. mongolicus*: *Astragalus membranaceus *Bge. var. *mongolicus *(Bge.) Hsiao; CG: calycosin-7-O-β-D-glucoside; TFs: transcription factors; *AmCAS*: cycloartenol synthase from *A. mongolicus*; CYP450: cytochrome P450 monooxygenase; GT: glycosyltransferase; ASI: astragaloside IV.

## Competing interests

The authors declare that they have no competing interests.

## Authors' contributions

PN and XL conceived and designed the study, and revised the manuscript. JC, XTW and YQX prepared samples, analyzed transcriptome data, drafted the manuscript, and performed experiment validation. YZ, YXL and JKC directed on the study and data analysis. All authors read and approved the final manuscript.

## Supplementary Material

Additional file 1 **List of annotated unigenes of *A. mongolicus *transcriptome as compared to public databases**. All assembled unigenes were annotated based on sequence similarity using four public databases, including Swiss-Prot (Sheet 1), CDD (Sheet 2), TrEMBL (Sheet 3) and PfamA (Sheet 4). Click here for file

Additional file 2 **COG classification of *A. mongolicus *transcriptome**. Phylogenetic classifications of the conserved domains of unigenes from *A. mongolicus *were analyzed by BLAST searching against COG database to predict and classify possible functions. In our study, 65,989 unigenes were classified into 24 COG categories. Click here for file

Additional file 3 **KEGG mapping of *A. mongolicus *transcriptome**. Unigenes from *A. mongolicus *were annotated against the KEGG database to investigate the metabolic pathways and related biological features. As a result, 13,057 transcripts were annotated with Enzyme Commission (EC) numbers and assigned to 339 reference canonical KEGG pathways. Click here for file

Additional file 4 **Transcription factors (TFs) identified from *A. mongolicus *transcriptome**. List of total TFs identified in *A. mongolicus *with RPKM value (Sheet 1). List of TF families with the number of related unigenes (Sheet 2). List of TFs expressed in different tissues (Unigenes with RPKM≥0.5 were considered as expressed. Sheet 3). List of the number of the four most abundant TF families expressed in different tissues (Unigenes with RPKM≥0.5 were considered as expressed. Sheet 4). Click here for file

Additional file 5 **The ORF sequence of *AmCAS *and primers used for PCR amplification in this study**. Specific primers used for the amplification of *AmCAS *sequence including CASF1, CASR1, CASF2, CASR2. Click here for file

Additional file 6 **CYP450 discovery**. List of total CYP450s identified in *A. mongolicus *with annotation and classification (Sheet 1). List of CYP450s co-expressed with *AmCAS *across three tissues (r>0.9, Sheet 2). Comparison of CYP450 clans among *A. mongolicus, Medicago truncatula *and *Arabidopsis thaliana *(Sheet 3). List of CYP450s expressed in different tissues (Unigenes with RPKM≥0.5 were considered as expressed. Sheet 4). Click here for file

Additional file 7 **GT discovery**. List of total GTs identified in *A. mongolicus *with annotation (Sheet 1). List of GTs co-expressed with *AmCAS *across three tissues (r>0.9, Sheet 2). Click here for file

## Declarations

Publication costs for this article were funded by the National Natural Science Foundation of China (Nos. 81373963).

This article has been published as part of *BMC Genomics *Volume 16 Supplement 7, 2015: Selected articles from The International Conference on Intelligent Biology and Medicine (ICIBM) 2014: Genomics. The full contents of the supplement are available online at http://www.biomedcentral.com/bmcgenomics/supplements/16/S7.

## References

[B1] XY Z Pharmacopoeia of the People's Republic of China 2005 2005 1 People's Medical Publishing House

[B2] Ma X Tu P Chen Y Zhang T Wei Y Ito Y Preparative isolation and purification of two isoflavones from Astragalus membranaceus Bge. var. mongholicus (Bge.) Hsiao by high-speed counter-current chromatography Journal of Chromatography A 2003 992 1 193 197 12735475 10.1016/s0021-9673(03)00315-7

[B3] Wu T Annie Bligh S Gu Lh Wang Zt Liu Hp Cheng Xm Branford-White CJ Hu Zb Simultaneous determination of six isoflavonoids in commercial Radix Astragali by HPLC-UV Fitoterapia 2005 76 2 157 165 10.1016/j.fitote.2004.11.006 15752625

[B4] Zhang Q Gao W Man S Chemical composition and pharmacological activities of astragali radix China journal of Chinese materia medica 2012 37 21 3203 3207 23397713

[B5] Pan H Wang Y Zhang Y Zhou T Fang C Nan P Wang X Li X Wei Y Chen J Phenylalanine ammonia lyase functions as a switch directly controlling the accumulation of calycosin and calycosin-7-O-β-D-glucoside in Astragalus membranaceus var. mongholicus plants Journal of experimental botany 2008 59 11 3027 3037 10.1093/jxb/ern152 18583351

[B6] Fan Y Wu DZ Gong YQ Zhou JY Hu ZB Effects of calycosin on the impairment of barrier function induced by hypoxia in human umbilical vein endothelial cells European journal of pharmacology 2003 481 1 33 40 10.1016/j.ejphar.2003.09.007 14637172

[B7] Lee YM Lee Yun-Mi Choi SI Choi Soo-Im Lee JW Lee Jae-Won Jung SM Jung Sun-Mi Park SM Park Sang-Min Heo TR Heo Tae-Ryeon Isolation of hyaluronidase inhibitory component from the roots of Astraglus membranaceus Bunge (Astragali Radix) Food Science and Biotechnology 2005 14 2 263 267

[B8] CHOI S PARK SR HEO TR Inhibitory effect of astragali radix on matrix degradation in human articular cartilage Journal of microbiology and biotechnology 2005 15 6 1258 1266

[B9] Pan H Fang C Zhou T Wang Q Chen J Accumulation of calycosin and its 7-O-β-d-glucoside and related gene expression in seedlings of Astragalus membranaceus Bge. var. mongholicus (Bge.) Hsiao induced by low temperature stress Plant cell reports 2007 26 7 1111 1120 10.1007/s00299-006-0301-8 17253088

[B10] Liu CJ Huhman D Sumner LW Dixon RA Regiospecific hydroxylation of isoflavones by cytochrome p450 81E enzymes from Medicago truncatula The Plant Journal 2003 36 4 471 484 10.1046/j.1365-313X.2003.01893.x 14617078

[B11] Liu CJ Deavours BE Richard SB Ferrer JL Blount JW Huhman D Dixon RA Noel JP Structural basis for dual functionality of isoflavonoid O-methyltransferases in the evolution of plant defense responses The Plant Cell Online 2006 18 12 3656 3669 10.1105/tpc.106.041376 PMC1785397 17172354

[B12] Du M WX Wang ZY Hu ZB Isolation and Identification of Genes Related to Astragaloside IV Differential Expressed Synthesis in Astragalus membranaceus Pharmaceutical Biotechnology 2005 12 2 76 80

[B13] Luo H Sun C Sun Y Wu Q Li Y Song J Niu Y Cheng X Xu H Li C Analysis of the transcriptome of Panax notoginseng root uncovers putative triterpene saponin-biosynthetic genes and genetic markers BMC genomics 2011 12 Suppl 5 S5 10.1186/1471-2164-12-S5-S5 22369100 PMC3287501

[B14] Dong H Jiang B Han Y GENG Y ZHAO Yq Transformation of Compound K from Saponins in Leaves of Panax notoginseng by Immobilized β-Glucanase Chin Herb Med 2010 2 1 41 47

[B15] Wu D Austin RS Zhou S Brown D The root transcriptome for North American ginseng assembled and profiled across seasonal development BMC genomics 2013 14 1 564 10.1186/1471-2164-14-564 23957709 PMC3751939

[B16] Wang S Li J Huang H Gao W Zhuang C Li B Zhou P Kong D Anti-hepatitis B virus activities of astragaloside IV isolated from radix Astragali Biological & pharmaceutical bulletin 2009 32 1 132 135 10.1248/bpb.32.132 19122295

[B17] Augustin JM Kuzina V Andersen SB Bak S Molecular activities, biosynthesis and evolution of triterpenoid saponins Phytochemistry 2011 72 6 435 457 10.1016/j.phytochem.2011.01.015 21333312

[B18] Kim O Bang K Jung S Kim Y Hyun D Kim S Cha S Molecular characterization of ginseng farnesyl diphosphate synthase gene and its up-regulation by methyl jasmonate Biologia plantarum 2010 54 1 47 53 10.1007/s10535-010-0007-1

[B19] Zhang WJ Hufnagl P Binder BR Wojta J Anti-inflammatory activity of astragaloside IV is mediated by inhibition of NF-qB activation and adhesion molecule expression Thromb Haemost 2003 90 5 904 914 14597987 10.1160/TH03-03-0136

[B20] Mardis ER The impact of next-generation sequencing technology on genetics Trends in genetics 2008 24 3 133 141 10.1016/j.tig.2007.12.007 18262675 PMC2680276

[B21] Ping J Wang Y Yu Y Li Y Li X Hao P A comparative analysis of tissue gene expression data from high-throughput studies Chinese Science Bulletin 2012 57 22 2920 2927 10.1007/s11434-012-5077-3

[B22] Vera JC Wheat CW Fescemyer HW Frilander MJ Crawford DL Hanski I Marden JH Rapid transcriptome characterization for a nonmodel organism using 454 pyrosequencing Molecular ecology 2008 17 7 1636 1647 10.1111/j.1365-294X.2008.03666.x 18266620

[B23] Shi CY Yang H Wei CL Yu O Zhang ZZ Jiang CJ Sun J Li YY Chen Q Xia T Deep sequencing of the Camellia sinensis transcriptome revealed candidate genes for major metabolic pathways of tea-specific compounds BMC genomics 2011 12 1 131 10.1186/1471-2164-12-131 21356090 PMC3056800

[B24] Wang XC Zhao QY Ma CL Zhang ZH Cao HL Kong YM Yue C Hao XY Chen L Ma JQ Global transcriptome profiles of Camellia sinensis during cold acclimation BMC genomics 2013 14 1 415 10.1186/1471-2164-14-415 23799877 PMC3701547

[B25] Liu XB Ma L Zhang AH Zhang YH Jiang J Ma W Zhang LM Ren WC Kong XJ High-Throughput Analysis and Characterization of Astragalus membranaceus Transcriptome Using 454 GS FLX PloS one 2014 16 5 e95831 24828103 10.1371/journal.pone.0095831 PMC4020759

[B26] Grabherr MG Haas BJ Yassour M Levin JZ Thompson DA Amit I Adiconis X Fan L Raychowdhury R Zeng Q Full-length transcriptome assembly from RNA-Seq data without a reference genome Nature biotechnology 2011 29 7 644 652 10.1038/nbt.1883 21572440 PMC3571712

[B27] Langmead B Trapnell C Pop M Salzberg SL Ultrafast and memory-efficient alignment of short DNA sequences to the human genome Genome Biol 2009 10 3 R25 10.1186/gb-2009-10-3-r25 19261174 PMC2690996

[B28] Tatusov RL Koonin EV Lipman DJ A genomic perspective on protein families Science 1997 278 5338 631 637 10.1126/science.278.5338.631 9381173

[B29] Jin J Zhang H Kong L Gao G Luo J PlantTFDB 3.0: a portal for the functional and evolutionary study of plant transcription factors Nucleic acids research 2014 42 D1 D1182 D1187 10.1093/nar/gkt1016 24174544 PMC3965000

[B30] Lorenzo CP Anahit G Irma RV F M-GJ R B-CJ L RD Genome-wide classification and evolutionary analysis of the bHLH family of transcription factors in Arabidopsis, poplar, rice, moss, and algae Plant Physiology 2010 3 10.1104/pp.110.153593 PMC2899937 20472752

[B31] Khanna R Huq E Kikis EA Al-Sady B Lanzatella C Quail PH A novel molecular recognition motif necessary for targeting photoactivated phytochrome signaling to specific basic helix-loop-helix transcription factors The Plant Cell Online 2004 16 11 3033 3044 10.1105/tpc.104.025643 PMC527196 15486100

[B32] Chinnusamy V Ohta M Kanrar S Lee Bh Hong X Agarwal M Zhu JK ICE1: a regulator of cold-induced transcriptome and freezing tolerance in Arabidopsis Genes & development 2003 17 8 1043 1054 10.1101/gad.1077503 12672693 PMC196034

[B33] Kiribuchi K Sugimori M Takeda M Otani T Okada K Onodera H Ugaki M Tanaka Y Tomiyama-Akimoto C Yamaguchi T *RERJ1*, a jasmonic acid-responsive gene from rice, encodes a basic helix-loop-helix protein Biochemical and biophysical research communications 2004 325 3 857 863 10.1016/j.bbrc.2004.10.126 15541369

[B34] Wei Z Yujin S Ljudmilla T Changbin C Ueli G Hong M Regulation of Arabidopsis tapetum development and function by DYSFUNCTIONAL TAPETUM1 (DYT1) encoding a putative bHLH transcription factor Development (Cambridge) 2006 16 10.1242/dev.02463 16831835

[B35] Nuno P Liam D Origin and diversification of basic-helix-loop-helix proteins in plants Molecular Biology and Evolution 2009 4 10.1093/molbev/msp288 PMC2839125 19942615

[B36] Qian W Tan G Liu H He S Gao Y An C Identification of a bHLH-type G-box binding factor and its regulation activity with G-box and Box I elements of the PsCHS1 promoter Plant cell reports 2007 26 1 85 93 16924502 10.1007/s00299-006-0202-x

[B37] R LS F HL L DS R WS Lc, a member of the maize R gene family responsible for tissue-specific anthocyanin production, encodes a protein similar to transcriptional activators and contains the myc-homology region Proceedings of the National Academy of Sciences of the United States of America 1989 18 10.1073/pnas.86.18.7092 PMC298000 2674946

[B38] Hu J Anderson B Wessler SR Isolation and characterization of rice R genes: evidence for distinct evolutionary paths in rice and maize Genetics 1996 142 3 1021 1031 8849907 10.1093/genetics/142.3.1021 PMC1207001

[B39] N N I D C J G P M C L L The TT8 gene encodes a basic helix-loop-helix domain protein required for expression of DFR and BAN genes in Arabidopsis siliques The Plant Cell 2000 10 10.1105/tpc.12.10.1863 PMC149125 11041882

[B40] Feller A Machemer K Braun EL Grotewold E Evolutionary and comparative analysis of MYB and bHLH plant transcription factors The Plant Journal 2011 66 1 94 116 10.1111/j.1365-313X.2010.04459.x 21443626

[B41] J PA D G U W A PP H S The regulatory c1 locus of Zea mays encodes a protein with homology to myb proto-oncogene products and with structural similarities to transcriptional activators The EMBO Journal 1987 12 10.1002/j.1460-2075.1987.tb02684.x PMC553820 3428265

[B42] Hartmann U Sagasser M Mehrtens F Stracke R Weisshaar B Differential combinatorial interactions of cis-acting elements recognized by R2R3-MYB, BZIP, and BHLH factors control light-responsive and tissue-specific activation of phenylpropanoid biosynthesis genes Plant molecular biology 2005 57 2 155 171 10.1007/s11103-004-6910-0 15821875

[B43] Katiyar A Smita S Lenka SK Rajwanshi R Chinnusamy V Bansal KC Genome-wide classification and expression analysis of MYB transcription factor families in rice and Arabidopsis BMC genomics 2012 13 1 544 10.1186/1471-2164-13-544 23050870 PMC3542171

[B44] Dubos C Stracke R Grotewold E Weisshaar B Martin C Lepiniec L MYB transcription factors in *Arabidopsis* Trends in plant science 2010 15 10 573 581 10.1016/j.tplants.2010.06.005 20674465

[B45] Chai G Hu R Zhang D Qi G Zuo R Cao Y Chen P Kong Y Zhou G Comprehensive analysis of CCCH zinc finger family in poplar (Populus trichocarpa) BMC genomics 2012 13 1 253 10.1186/1471-2164-13-253 22708723 PMC3427045

[B46] Dong W Yinghui G Changai W Guodong Y Yingying L Chengchao Z Genome-wide analysis of CCCH zinc finger family in Arabidopsis and rice BMC Genomics 2008 1 10.1186/1471-2164-9-44 PMC2267713 18221561

[B47] I CS R HT B-H M H CJ H CB R PS Alleviation of osteoarthritis by calycosin-7-O-beta-D-glucopyranoside (CG) isolated from Astragali radix (AR) in rabbit osteoarthritis (OA) model Osteoarthritis and Cartilage 2007 9 10.1016/j.joca.2007.02.015 17408983

[B48] M MS K HT K GR K BH Phytoestrogens and breast cancer prevention: possible mechanisms of action Environmental Health Perspectives 2008 4 10.1289/ehp.10538 PMC2291001 18414622

[B49] Yu O McGonigle B Metabolic Engineering of Isoflavone Biosynthesis Advances in Agronomy 2005

[B50] Werck-Reichhart D Batard Y Kochs G Lesot A Durst F Monospecific polyclonal antibodies directed against purified cinnamate 4-hydroxylase from Helianthus tuberosus (immunopurification, immunoquantitation, and interspecies cross-reactivity) Plant physiology 1993 102 4 1291 1298 10.1104/pp.102.4.1291 8278549 PMC158918

[B51] Jae-youl J Mangai KG Ji-young P Won-jin K Hyun-soon K Bong-sik Y Hyouk J Jae-heung J An overexpression of chalcone reductase of Pueraria montana var. lobata alters biosynthesis of anthocyanin and 5'-deoxyflavonoids in transgenic tobacco Biochemical and Biophysical Research Communications 2003 1 10.1016/s0006-291x(03)00344-9 12646206

[B52] Xu RY Nan P Yang Y Pan H Zhou T Chen J Ultraviolet irradiation induces accumulation of isoflavonoids and transcription of genes of enzymes involved in the calycosin-7-O-β-d-glucoside pathway in Astragalus membranaceus Bge. var. mongholicus (Bge.) Hsiao Physiologia plantarum 2011 142 3 265 273 10.1111/j.1399-3054.2011.01474.x 21438882

[B53] Gillissen B Bürkle L André B Kühn C Rentsch D Brandl B Frommer WB A new family of high-affinity transporters for adenine, cytosine, and purine derivatives in Arabidopsis The Plant Cell Online 2000 12 2 291 300 10.1105/tpc.12.2.291 PMC139765 10662864

[B54] Kim YB Thwe AA Li X Tuan PA Zhao S Park CG Lee JW Park SU Accumulation of Flavonoids and Related Gene Expressions in Different Organs of Astragalus membranaceus Bge Applied Biochemistry and Biotechnology 2014 1 10 24903957 10.1007/s12010-014-1004-1

[B55] Dubey VS Bhalla R Luthra R An overview of the non-mevalonate pathway for terpenoid biosynthesis in plants Journal of biosciences 2003 28 5 637 646 10.1007/BF02703339 14517367

[B56] Lichtenthaler HK Schwender J Disch A Rohmer M Biosynthesis of isoprenoids in higher plant chloroplasts proceeds via a mevalonate-independent pathway Febs Letters 1997 400 3 271 274 10.1016/S0014-5793(96)01404-4 9009212

[B57] Wolff M Seemann M Tse Sum Bui B Frapart Y Tritsch D Estrabot AG Rodríguez-Concepción M Boronat A Marquet A Rohmer M Isoprenoid biosynthesis via the methylerythritol phosphate pathway: the (*E*)-4-hydroxy-3-methylbut-2-enyl diphosphate reductase (LytB/IspH) from *Escherichia coli* is a [4Fe-4S] protein FEBS letters 2003 541 1 115 120 12706830 10.1016/s0014-5793(03)00317-x

[B58] Vincken JP Heng L de Groot A Gruppen H Saponins, classification and occurrence in the plant kingdom Phytochemistry 2007 68 3 275 297 10.1016/j.phytochem.2006.10.008 17141815

[B59] CHIOU Wf ZHANG Jt Comparison of the pharmacological effects of Panax ginseng and Panax quinquefolium Acta Pharmacologica Sinica 2008 29 9 1103 1108 10.1111/j.1745-7254.2008.00868.x 18718179

[B60] Han JY Kwon YS Yang DC Jung YR Choi YE Expression and RNA interference-induced silencing of the dammarenediol synthase gene in Panax ginseng Plant and cell physiology 2006 47 12 1653 1662 10.1093/pcp/pcl032 17088293

[B61] Y.H. L Shi. S H.Q. Z Y.D. Z B. X 2D NMR study on cycloartane triterpenoids from Astragalus membranaceus var. mongholicus Journal of China Pharmaceutical University 2008 39 1 15 19

[B62] Phillips DR Rasbery JM Bartel B Matsuda S Biosynthetic diversity in plant triterpene cyclization Current opinion in plant biology 2006 16 3 305 314 16581287 10.1016/j.pbi.2006.03.004

[B63] Corey E Matsuda S Bartel B Isolation of an Arabidopsis thaliana gene encoding cycloartenol synthase by functional expression in a yeast mutant lacking lanosterol synthase by the use of a chromatographic screen Proceedings of the National Academy of Sciences 1993 90 24 11628 11632 10.1073/pnas.90.24.11628 PMC48037 7505443

[B64] Nelson DR Schuler MA Paquette SM Werck-Reichhart D Bak S Comparative genomics of rice and Arabidopsis. Analysis of 727 cytochrome P450 genes and pseudogenes from a monocot and a dicot Plant Physiology 2004 135 2 756 772 10.1104/pp.104.039826 15208422 PMC514113

[B65] Masaaki S Masaki H Yuji K Hiroaki H Tetsuo K Yutaka E Identification of beta-amyrin and sophoradiol 24-hydroxylase by expressed sequence tag mining and functional expression assay The FEBS Journal 2006 5 10.1111/j.1742-4658.2006.05120.x 16478469

[B66] Hikaru S Kiyoshi O Satoru S Masaharu M Toshiyuki O Hiroshi S Tomoyoshi A Toshio A Kazuki S Toshiya M Licorice beta-amyrin 11-oxidase, a cytochrome P450 with a key role in the biosynthesis of the triterpene sweetener glycyrrhizin Proceedings of the National Academy of Sciences of the United States of America 2008 37 10.1073/pnas.0803876105 PMC2532699 18779566

[B67] Lahoucine A V HD A FM W SL W BJ A DR Genomics-based selection and functional characterization of triterpene glycosyltransferases from the model legume Medicago truncatula The Plant Journal 2005 6 10.1111/j.1365-313X.2005.02344.x 15743451

[B68] WANG YP LI XY SONG CQ HU ZB Effect of astragaloside F on T, B lymphocyte proliferation and peritoneal macrophage function in mice1 Acta Pharmacol Sin 2002 23 3 263 266 11918853

[B69] Luo H Dai R Li Y Nuclear cardiology study on effective ingredients of Astragalus membranaceus in treating heart failure Chinese journal of integrated traditional and Western medicine 1995 15 12 707 709 8732134

[B70] Luo Y Qin Z Hong Z Zhang X Ding D Fu JH Zhang WD Chen J *Astragaloside IV* protects against ischemic brain injury in a murine model of transient focal ischemia Neuroscience letters 2004 363 3 218 223 10.1016/j.neulet.2004.03.036 15182947

[B71] Zhou J Fan Y Kong J Wu D Hu Z Effects of components isolated from Astragalus membranaceus Bunge on cardiac function injured by myocardial ischemia reperfusion in rats China journal of Chinese materia medica 2000 25 5 300 302 12512456

[B72] Bak S Beisson F Bishop G Hamberger B Höfer R Paquette S Werck-Reichhart D Cytochromes P450 The Arabidopsis Book/American Society of Plant Biologists 2011 16 10.1199/tab.0144 PMC3268508 22303269

[B73] Bernhardt R Cytochromes P450 as versatile biocatalysts Journal of biotechnology 2006 124 1 128 145 10.1016/j.jbiotec.2006.01.026 16516322

[B74] Nelson D Werck-Reichhart D A P450-centric view of plant evolution The Plant Journal 2011 66 1 194 211 10.1111/j.1365-313X.2011.04529.x 21443632

[B75] K AG Shigeru T Oksoo H Hitoshi I Randeep R Rice octadecanoid pathway Biochemical and Biophysical Research Communications 2004 1 10.1016/j.bbrc.2004.03.020 15047141

[B76] K HR Stefania DD Angelo S Plant cytochrome CYP74 family: biochemical features, endocellular localisation, activation mechanism in plant defence and improvements for industrial applications ChemBioChem 2009 7 10.1002/cbic.200800633 19322850

[B77] Li T Valeria M Joonyul K Maria ML Dean D The Arabidopsis LUT1 locus encodes a member of the cytochrome p450 family that is required for carotenoid epsilon-ring hydroxylation activity PROCEEDINGS OF THE NATIONAL ACADEMY OF SCIENCES OF THE UNITED STATES OF AMERICA 2004 1 10.1073/pnas.2237237100 PMC314197 14709673

[B78] M TE Shozo F Hideharu S Yukihisa S Suguru T Shigeo Y A DM I TQ M NM CYP72B1 inactivates brassinosteroid hormones: an intersection between photomorphogenesis and plant steroid signal transduction Plant Physiology 2003 4 10.1104/pp.103.030882 PMC300720 14605216

[B79] J BG Csaba K Brassinosteroids and plant steroid hormone signaling The Plant Cell 2002 10.1105/tpc.001461 PMC151250 12045272

[B80] Franck P Fred B Cytochrome P450 metabolizing fatty acids in plants: characterization and physiological roles The FEBS Journal (Online) 2010 2 10.1111/j.1742-4658.2010.07948.x 21156024

