# Supplementary material for: Global transcriptome analysis profiles metabolic pathways in traditional herb Astragalus membranaceus Bge. var. mongolicus (Bge.) Hsiao
Source: BMC Genomics. 2015 Jun 11;16(Suppl 7):S15. doi: 10.1186/1471-2164-16-S7-S15 (PMC4474414; doi:10.1186/1471-2164-16-S7-S15)
Supplement: Additional file 5 — The ORF sequence of AmCAS and primers used for PCR amplification in this study. Specific primers used for the amplification of AmCAS sequence including CASF1, CASR1, CASF2, CASR2. [file 1471-2164-16-S7-S15-S5.docx]

**ORF Sequence of CAS: Total 2274bp**

ATGTGGAAGTTGAAGATTGCGGAGGGAGGGAGTCCATGGCTACGAACATTGAACAATCACGTTGGGAGACAAGTTTGGGAGTTCGATCCTAAACTTGGATCGCCAGAAGATCTCTTGGAGATTGAAAATGCTCGTCAAAATTTTCACAATAATCGATTTACTCAACAACATAGCGCTGATTTACTTATGCGCTTTCAGTTTGCAAAAGAGAACCCAATGAACGAAGTCTTATCCAAAGTGAAAGTCAAAGATATTGAGGATGTAACTGAAGAAACTGTGACAACAACATTAAGAAGGGCGTTAAACTTCCATTCAACACTCCAGAGTCATGATGGACACTGGTCAGGAGATTATGGAGGTCCCATGTTTCTTATGCCTGGCTTGATAATTGTCCTCTCAATCACTGGGGCTCTGAATGCAGTCTTGACAGAAGAGCATAGAAAGGAAATGTGCCGTTACCTCTATAATCATCAGAACAAGGATGGTGGGTGGGGTTTGCATATTGAAGGTCCAAGCACCATGTTTGGCTCTGTCTTATCTTATGTTTCTCTGAGATTGCTTGGTGAGGGACCTAATGACGGACAAGGGGAAATGGAGAAGGGTCGTGACTGGATTCTTGGGCATGGTGGTGCTACTTTTATAACATCATGGGGGAAGATGTGGCTTTCAGTACTTGGAGTGTATGAATGGTCTGGAAATAATCCCCTACCTCCTGAGATATGGCTCCTTCCATACATGCTTCCATTTCATCCAGGAAGGATGTGGTGTCACTGTCGGATGGTCTATTTGCCAATGTCATACTTATATGGCAAGAGGTTTGTTGGCCCAATCACACCAACAATATTATCGTTGAGAAAAGAGCTTTATACCACACCATATCATGATATAGACTGGAATCAGTCTCGAAATTTGTGTGCAAAGGAGGACTTGTACTATCCTCACCCACTCGTGCAAGATATTCTCTGGGCATCTCTACACAAGGTTATTGAGCCCATTTTGATGCATTGGCCTGCCAAAAAGTTGAGAGAAATGGCTACTAGGACTGCAATTGAGCATATACATTATGAAGATGAGAATACTCGATATATTTGCATAGGTCCTGTTAATAAGGTGTTAAATATGCTTTGCTGTTGGGTGGAAGATCCAAATTCAGAGGCCTTCAAGTTGCATCTTCCAAGGATCCATGATTATCTATGGGTTGCAGAAGATGGCATGAAAATGCAGGGATACAACGGGAGTCAACTATGGGATACTGCTTTTGCTGCCCAAGCAATTATTTCAACTAACCTCATTGAAGAGTATGGTCCAACTCTAAGAAAAGCTCATACATACATTAAGAATTCACAGGTTTTAGAAGATTGTCCAGGTGATCTTAGTAAATGGTACCGTCACATTTCAAAAGGGGCATGGCCTTTCTCGACTGCAGACCATGGATGGCCAATTTCTGACTGCACAGCTGAAGGACTGAAAGCTGTTCTTTTGCTATCTAAAATTCCACCAGAGATTGTTGGTGAGCCAGTGGATGCAAAGCGTTTATATGACTCTGTAAATGTCATTCTCTCGTTACAGAATGAAGATGGTGGCTATGCAACATATGAGCTCAAACGATCTTATGACTGGTTGGAGTTAATCAATCCTGCTGAAACTTTTGGTGACATCGTTATTGATTATCCTTATGTGGAATGTACCTCAGCAGCAATTCAAGCTTTGACATCATTTATGAAACTATATCCCGGGCATCGTCGAGAAGAAATACAATGCTGCATTGATAAAGCTGTTGCCTATATTGAAAAAATACAAGCTTCGGATGGTTCATGGTATGGTTCTTGGGCAGTTTGCTTCACTTATGGCACTTGGTTTGGGGTCAAAGGGCTGATTGCTGCTGGAAAGAGTTACAATAATTGCTCAAGCATCCGCAAAGCTTGTGAATTCCTTCTGTCTAAACAGCTCCCATCTGGTGGCTGGGGAGAGAGTTATCTGTCATGTCAAAATAAGGTTTATTCAAATTTGGAAGGCAACAGGTCCCATGTGGTAAACACTGGGTGGGCTATGCTGGCTCTCATTGATGCTGAGCAGGCAAAGAGAGATCCAATGCCACTGCATCGTGCAGCTCAATACTTGATAAATTCCCAAATGGAGAATGGTGACTTTCCGCAACAGGAAATAATGGGAGTCTTCAACAAGAATTGCATGATCACATACGCTACATACAGAAACATATTCCCCATTTGGGCGTTGGGAGAATACCGTGATCGTGTATTGCAGGCCCGTTAA

| **Primer** | **Sequence** | **length** | **Product** |
| --- | --- | --- | --- |
| **CASF1** | TTCGTTATTATTACTGCTACCATTTCTC | 28 | **CASA**  1324 bp |
| **CASR1** | TCCCATAGTTGACTCCCGTTG | 21 |  |
| **CASF2** | TCTCTGGGCATCTCTACACAAGGT | 24 | **CASB**  1425 bp |
| **CASR2** | AACTCAAAGCAACTCATTGGAGC | 23 |  |
